# Interaction of Bis-(sodium-sulfopropyl)-Disulfide and Polyethylene Glycol on the Copper Electrodeposited Layer by Time-of-Flight Secondary-Ion Mass Spectrometry

**DOI:** 10.3390/molecules28010433

**Published:** 2023-01-03

**Authors:** Robert Mroczka, Agnieszka Słodkowska, Agata Ładniak, Agnieszka Chrzanowska

**Affiliations:** 1 Laboratory of X-ray Optics, Department of Chemistry, Institute of Biological Sciences, Faculty of Medicine, The John Paul II Catholic University of Lublin, Konstantynów 1J, 20-708 Lublin, Poland; 2 Institute of Chemical Sciences, Faculty of Chemistry, Maria Curie-Sklodowska University, Maria Curie-Sklodowska Sq. 3, 20-031 Lublin, Poland

**Keywords:** copper electrodeposition, SPS (bis-(sodium-sulfopropyl)-disulfide), polyethylene glycol, TOF-SIMS, cyclic voltammetry

## Abstract

The interactions of the functional additives SPS (bis-(sodium-sulfopropyl)-disulfide) and polyethylene glycol (PEG) in the presence of chloride ions were studied by time-of-flight secondary-ion mass spectrometry (TOF-SIMS) in combination with cyclic voltammetry measurements (CV). The PEG, thiolate, and chloride surface coverages were estimated and discussed in terms of their electrochemical suppressing/accelerating abilities. The conformational influence of both the gauche/trans thiolate molecules, as well as around C-C and C-O of PEG, on the electrochemical properties were discussed. The contribution of the hydrophobic interaction of -CH_2_-CH_2_- of PEG with chloride ions was only slightly reduced after the addition of SPS, while the contribution of Cu-PEG adducts diminished strongly. SPS and PEG demonstrated significant synergy by significant co-adsorption. It was shown that the suppressing abilities of PEG that rely on forming stable Cu-PEG adducts, identified in the form C_2_H_4_O_2_Cu^+^ and C_3_H_6_OCu^+^, were significantly reduced after the addition of SPS. The major role of thiolate molecules adsorbed on a copper surface in reducing the suppressing abilities of PEG rely on the efficient capture of Cu^2+^ ions, diminishing the available copper ions for the ethereal oxygen of PEG.

## 1. Introduction

Copper electrodeposition plays an important role in manufacturing micro- and nanodevices [1,2,3,4]. In order to control electrodeposition processes at the nano- and microscale to fulfill demanding requirements from the industrial side, appropriate additives in the electroplating bath have to be applied [3,5,6,7].

For nanoscale processes when appropriate fulfilling sub-micrometer features are required, polyethylene glycol (PEG) is added to the electroplating bath [8,9,10,11] in order to suppress the copper electrodeposition. However, the exact mechanism of polyethylene glycol is still not fully understood. In one model, the interaction of PEG in the presence of chloride ions relies on forming a stable PEG-Cu^+^-Cl^−^ complex when Cu^+^ ions are bonded to two ethereal atoms of PEG and play the role of binding agent to Cl^−^ ions that form an adlayer directly on the copper electrode [12,13,14]. However, neither Cu^+^ nor Cu^2+^ ions are required for the efficient bonding of PEG onto a copper substrate [15]. On the other hand, the existence of complexes of Cu^2+^ with PEG [16] and Hg^2+^ with PEG [17,18,19] have been reported. Moreover, in the second model of copper electrodeposition with the assistance of PEG, the chloride adlayer played a substrate for the hydrophobic parts of PEG through nonhydrogen bonded water [20,21]. Complexes of PEG(Na^+^)(Cu^+^) were also identified on the copper electrodeposited surface by the authors of this work [22].

The situation is even more complicated when a third additive such as SPS, playing an accelerating role, is included in the composition of the electroplating bath [5,9,23,24,25,26,27,28,29]. The proposed accelerating mechanism of action of SPS [3,30] relies on the formation of stable complexes in the form of Cu(I)Cl-MPS- that stabilize the Cu^+^. On the contrary, some spectroscopic studies (e.g., shell-isolated nanoparticle-enhanced Raman spectroscopy (SHINERS) [31,32,33,34,35,36]) suggest that thiolate molecules in gauche conformation are efficient in the partial dehydration of Cu^2+^ and in the next step after reducing Cu^2+^ to Cu^+^ transport Cu^+^ ions to the chloride adlayer [37].

Taking into account the above, it is obvious that the exact mechanism of interaction of PEG-SPS-Cl is very complex and requires appropriate spectroscopic data for a more accurate interpretation of the role of individual additives for copper electrodeposition.

Accordingly, traditional electrochemical techniques. such as cyclic voltammetry (CV) and the extracted CV curves’ Tafel slopes, are not sufficient tools for the determination and evaluation of the mechanistic model of kinetics and interaction between additives on a copper electrode at the molecular level [38,39]. Some authors, based on FE-SEM micrographs, reported that the injection of 1 ppm of SPS into PEG/Cl solutions led to the complete removal of PEG from the copper surface [6]. However, no spectroscopic evidence was provided to support this hypothesis. Moreover, the sample rinsing method after electrodeposition was not explained, while it is obvious that this step of preparation of a sample for examination is very crucial. On the other hand, the competitive adsorption of SPS with PEG results in a progressive displacement, reduced co-adsorption, or disruption of PEG-Cl-inhibiting film [28,40,41,42,43]. Broekmann et al. [24], based on STM measurements, reported that the adsorption of MPS molecules within the Cl adlayer stimulates the Cl adlayer to be deactivated for PEG adsorption due to the spherical hindrance of large sulfonate groups pointed towards the solution. On the other hand, the experiments were carried out under nonreactive conditions at relatively high SPS concentrations (1 mM corresponds to 154 ppm) with chloride ions (concentration of 350 ppm) and without copper ions in the examined solution. Due to the fact of this reason, the received results could significantly differ from the real electroplating conditions. 

The electrochemistry of PEG-Cl-SPS has been studied thoroughly [5,44,45,46]. Unfortunately, there is still a limited number of appropriate experiments oriented toward the combination of cyclic voltammetry experiments with a modern spectroscopic technique that is useful for resolving the interaction of PEG/SPS/Cl. 

However, increasingly, electrochemical impedance spectroscopy (EIS) [47], electrochemical quartz crystal microbalance (ECQ) [7,48,49], atomic force microscopy (AFM) [7,9,26,44,50], and scanning tunneling microscopy (STM) [24,51] are served as complementary techniques. Unfortunately, ECQ or STM requires removing Cu^2+^ ions from the electroplating bath due to the difficulties residing in the methodology of the technique, e.g., copper electrodeposition does not allow to record the weight of the adsorbates by ECQ, while in the case of STM, higher amounts of Cu^2+^ and higher current densities do not allow for the scanning tip to work properly. Moreover, the most powerful spectroscopic techniques, such as SERS [12,48,49] or its SEIRAS variant, demonstrate similar drawbacks. For ex situ measurements, the SERS technique suffers from copper sulfate crystallization and surface film moving. Moreover, variations of roughness during copper electrodeposition stimulate different SERS signals from the copper substrate, which are very difficult to eliminate and control. Due to the fact of this reason, SERS investigations were limited to the solution without Cu^2+^ ions. On the contrary, to avoid some of these problems, in situ measurements by SHINERS were carried out. However, this variant exhibits a significantly lower detection limit and requires very high concentrations of additives [31,32,33,34,35,36]. Interestingly, recently, SHINERS studies were combined with X-ray photoelectron spectroscopy (XPS) for mapping the coverage and state of surfactant additives during electrochemical processes [52].

However, there are no successful attempts that allow for investigating the electrochemistry of PEG-Cl-SPS by SERS [26] while examining the interactions of methanesulfonic acid (MSA) and PEG [48]. The MSA was used as a substitution for sulfuric acid with a concentration 0.12 M, which is largely higher than a routinely applied concentration of SPS (on the order of a few ppm).

Additionally, quantum chemistry calculations, as complementary and supporting studies to experimental techniques based on density functional theory (DFT), were also applied [12,31,51,53,54,55].

Mass spectroscopy such as electrospray ionization (ESI) can supply information concerning the byproducts of copper electrodeposition, such as Cu(I)SC_3_H_6_SO_3_^−^ [54]. However, an additional complementary mass spectrometry technique that provides molecular analysis of thin films on a copper surface is required. Time-of-flight secondary-ion mass spectrometry (TOF-SIMS) fulfills those expectations and was applied for electrodeposited copper surface analysis [22,50,56,57,58,59,60,61,62,63], serving very high detection limits (in the range of ppm) and a high lateral resolution for molecular imaging (below 1 µm).

Recently, we showed that static TOF-SIMS combined with cyclic voltammetry measurements can be successfully applied in molecular studies of polyethylene glycol in the presence of chloride ions during copper electrodeposition [22] and for investigations of MPS/SPS interactions with chloride ions [63]. In the latter study [63], it was shown that thiolate molecules were incorporated into the copper deposit. Moreover, the influence of thiolate and chloride surface coverage and the ratio of gauche/trans thiolate conformations on the accelerating abilities was studied. Since the TOF-SIMS method cannot be used for in situ measurements due to the ultrahigh vacuum conditions in the analysis chamber, we designed and optimized an experimental condition that allowed us to evaluate the chemistry of the copper surface after electrodeposition. Instead of a flat substrate, which is routinely applied as a working electrode, nitinol wire was used and withdrawn at a controlled, constant speed from the electroplating bath during copper electrodeposition [22,63]. In this manner, the appropriate positions along the wire strictly corresponded to the applied overpotential points on the CV curve. Moreover, due to the high concentration of copper sulfate in the solution, the method of rinsing the samples after the CV experiment was optimized to avoid, on the one hand, the crystallization of the copper sulfate and, on the other, to preserve a maximum amount of adsorbate material on the surface. More details can be found in our previous papers [22,63] and in Section 3.

In the present work, we focused our efforts on the examination of the interaction between SPS and PEG in the presence of chloride ions to explore the role of SPS in decreasing the suppressing abilities of PEG at the molecular level under real electrochemical conditions using a combination of CV and TOF-SIMS. According to our best knowledge, no similar attempts have been undertaken with the SIMS method and in SERS/SHINERS studies.

## 2. Results and Discussion

### 2.1. Cyclic Voltammetry Measurements

**Figure 1:**
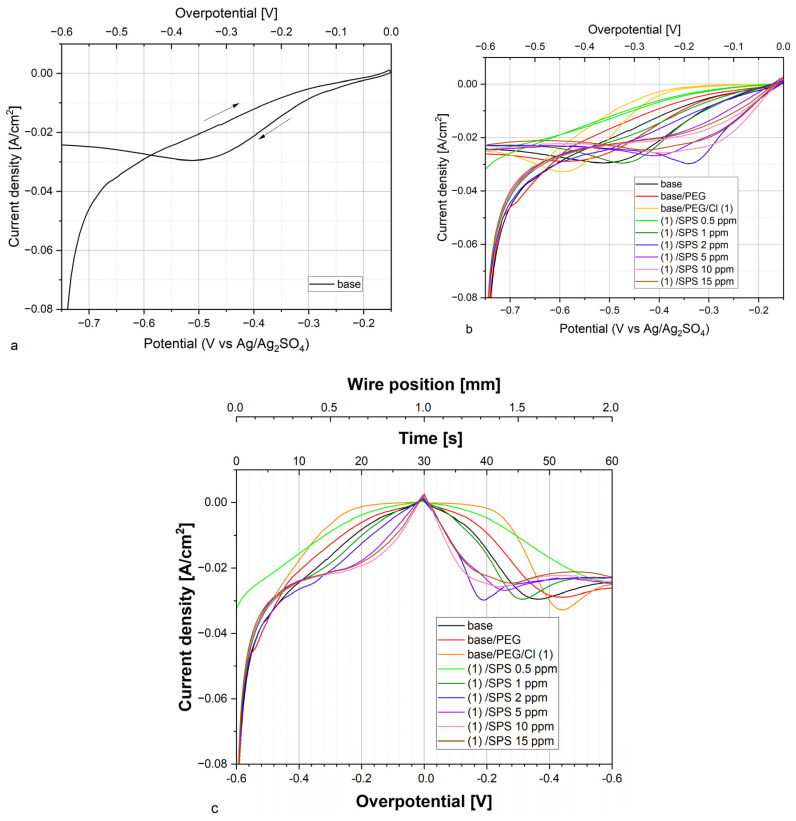
(**a**) Cyclic voltammetry curve recorded for the base electrolyte. (**b**) Cyclic voltammetry curves recorded for the base electrolyte and after the addition of 400 ppm of PEG8000, 30 ppm of Cl, and SPS at concentrations of 0.5, 1, 2, 5, 10, and 25 ppm. Due to the very stable value of OCP (Figure S1, Supplementary Materials) that equaled −151mV +/− 2 mV for all samples’ overpotential is also shown in the top axis. (**c**) CV curves shown as a function of time. The time from 0 to 30 s and the overpotential from −0.6 to 0 V (OCP) corresponds to the forward scan, while the time from 30 to 60 s and the overpotential from 0 (OCP) to −0.6 V corresponds to the reverse scan that was shown in Figure 1a,b, respectively. The wire position from 0 to 1 mm corresponds to the forward scan from −0.6 V to OCP (overpotential 0 V), while the wire position from 1 to 2 mm corresponds to the reverse scan from OCP to −0.6 V.

Figure 1a depicts the cyclic voltammetry curve for the base solution (B). The potential was recorded by means of sulfate reference electrode Ag/Ag_2_SO_4_ to avoid the risk of the leakage of the chloride ions. Figure 1b exhibits a cyclic voltammetry curve for the base solution (B), base + 400 ppm PEG (B + P), and (B + P) + 30 ppm of Cl^−^ ions (B + P + Cl) after the addition of 0.5, 1, 2, 5, 10, and 15 ppm SPS. The OCP (open circuit potential) was very stable for all samples (Figure S1, Supplementary Materials) and was varied from −148 mV for the base electrolyte to −153.7 mV for the PEG/Cl sample. The average value of the OCP was −151 mV +/− 2 mV. Due to the fact of this reason, the axis with overpotential could be well matched with the potential recorded by the sulfate electrode and was added to the top of Figure 1. Figure 1c demonstrates CV curves that show the current density versus the overpotential (bottom axis) and time (top axis). Moreover, the position of the wire that corresponded to the length of the wire that was withdrawn from the electroplating bath during the recording of the CV curves is shown in the top axis. In this manner, the selected position of the wire corresponded to the selected overpotential or potential recorded by the reference sulfate electrode. For example, the position of the wire ranged from 0 to 1 mm, corresponding to the forward scan (overpotential: −0.6 to 0 (OCP); time: 0 to 30 s), while the wire position from 1 to 2 mm corresponded to the reverse scan (overpotential: 0 to −0.6 V; time: 30 to 60 s). The velocity of 34 µm/s of wire withdrawing from the solution was matched with a CV scan rate 20 mV/s. In this way, the first 1 mm of wire that was withdrawn over 30 s corresponded to the CV forward scan, while the second 1 mm of wire (positions: 1 to 2 mm) was matched to the reverse scan (Figure 1c).

Figure 1c depicts the effect of the injection of additives into the base solution. After the addition of PEG, minor suppressing abilities were observed. The next portion of chloride ions significantly inhibited copper deposition in the wide range of overpotential from approximately −0.45 V for the forward scan to −0.3 V for the reverse scan. The addition of SPS caused a gradual decrease in the suppressing abilities of the PEG + Cl layer as a function of the SPS concentration. The greatest accelerating abilities were observed after the addition of 10 and 15 ppm SPS for the forward scan, while the accelerating abilities at 15 ppm of SPS were reduced for the reverse scan in comparison to the solution containing 10 ppm of SPS. On the other hand, the greatest increase in accelerating abilities was observed when the SPS concentration increased from 0.5 to 1 ppm. 

To evaluate quantitively the differences between the samples, the exchange current densities *j*_0,1_ and *j*_0,2_ were calculated. The exchange current density *j*_0,1_, which determines the reduction of Cu^+^ to metallic Cu, was calculated for very low overpotentials of less than −10 mV both for the forward and reverse scans. The data shown in Figure 1c and Figure S2 (for the base solution, Supplementary Materials) were fitted to a simplified version of the Butler–Volmer Equation (1) that can be limited to the first term of the Taylor series for very low overpotentials, similarly as it was applied by other researchers [37] and proposed in a classical electrochemical book [64]:

(1) $$j\;=\;j_{0,1}\;\left(\frac{nF\eta }{RT}\right),$$

where *j*_0,1_ denotes the exchange current density for the Cu^+^ to Cu reduction; *n* is the number of transferred electrons (*n* = 1); *F* is the Faraday constant (C/mol); *ƞ* is the overpotential (V); *R* is the gas constant (J/(mol·K)); and *T* is the temperature (K).

The neglection of the higher order terms in the general Butler–Volmer equation for two electron reduction steps for Cu^2+^ reduction assumes that the transfer coefficient for Cu^+^ reduction is zero [37]. On the other hand, linear approximation for the Butler–Volmer equation is routinely applied for low overpotentials [64]. An example of a fitting procedure for the forward scan for a base solution that allows us to obtain *j*_0,1_ is shown in Figure S2 (Supplementary Materials).

Furthermore, exchange current density *j*_0,2_, which corresponds to the reduction of Cu^2+^ to Cu^+^, was obtained by fitting the linear part of the Tafel equation for cathodic processes that were valid for the high negative values of the overpotentials (*ƞ* < −0.1 V):

(2) $$\eta \;=\;a\;-\;blog\;\left(-\;j_{0,2}\right),$$

where *ƞ* is the overpotential

(3) $$a\;=\;2.303\;\frac{RT}{\alpha_{0,2}F\;}\log\;j_{0,2}$$

(4) $$b\;=\;2.303\;\frac{RT}{\alpha_{0,2}F}$$

and α_0,2_ is the transfer charge coefficient for Cu^2+^ reduction to Cu^+^.

We noticed that the linear part of Tafel in Equation (2) can be observed for the overpotentials of approximately −0.18 V for the forward as well as reverse scans for all of the analyzed samples. The linear regression that was carried out for the calculation of exchange current density *j*_0,2_ for the base solution is shown in Figure S3 (Supplementary Materials). We chose the same overpotential region of the Tafel equation (approximately −0.18 V) to be able to compare the *j*_0,2_ for the forward scan and reverse scan. In the linear regression, the Tafel slope (*b*) was assigned as the slope, while the Tafel parameter *a* was assigned as the intercept (Figure S3, Supplementary Materials).

**Figure 2:**
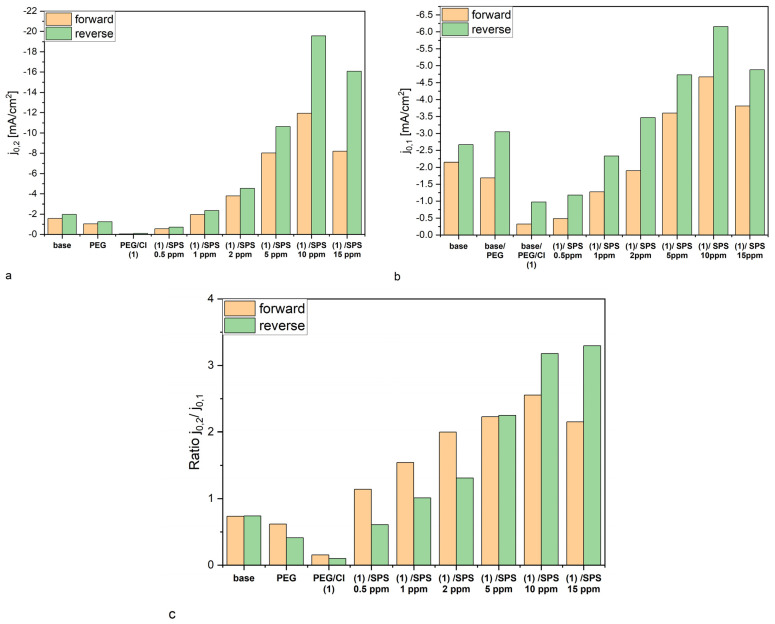
Exchange current densities *j*_0,1_ (**b**) and *j*_0,2_ (**a**) and the ratio *j*_0,2_/*j*_0,1_ (**c**) for the base, base/PEG, base/PEG/Cl, and base/PEG/Cl/SPS at concentrations 0.5, 1, 2, 5, 10, and 15 ppm. The orange bars correspond to the forward scan and the green bars to the reverse scan.

The corresponding exchange current densities *j*_0,2_ and *j*_0,1_ are depicted in Figure 2a,b. It is widely assumed that the reduction of Cu^+^ to the metallic Cu is based on the inner-sphere mechanism [38], while the reduction of Cu^2+^ to Cu^+^ is determined by ion migration reactions driven by the reorganization of solvent and surface coverage [65].

It is clearly seen that exchange current density *j*_0,2_ (Figure 2a) was strongly suppressed by the addition of chloride ions to the base + PEG solution, while after the addition of PEG to the base solution, only a minor suppressing effect was observed. The addition of SPS strongly increased the exchange current density *j*_0,2_, while the changes were not linearly proportional to the SPS concentration for the forward scan (see Figure S4, Supplementary Materials), demonstrating the maximum value for 10 ppm SPS. Specifically, after increasing the SPS concentration from 10 to 15 ppm exchange current density *j*_0,2_ was reduced and demonstrated a similar value as observed at 5 ppm SPS. On the contrary, for the reverse scan, the increase in the exchange current density *j*_0,2_ was roughly proportional to the SPS concentration (Figure S4, Supplementary Materials), while only at 15 ppm could we observe the diminishment of the exchange current density but less distinctly than it took place for the forward scan. The differences in exchange current densities *j_0.2_* showed a significant hysteresis between the forward and reverse scans (see Figure 1b), determined by the irreversible type of reaction response at the electrode. The accelerating abilities for the whole range of concentrations of SPS were significantly higher on the desorption side (i.e., reverse scan). For low overpotentials, exchange current density *j*_0,1_ demonstrated similar behavior for the reverse scan and forward scan (Figure 2b and  Figure S5 in the Supplementary Materials), while for the reverse scan, we observed higher values of *j*_0,1_, similar to what was observed for *j*_0,2_. On the contrary to *j*_0,2_ we did not observe linearity in the range of SPS concentrations from 0.5 to 10 ppm for the reverse scan (Figure S5, Supplementary Materials).

However, the ratio *j*_0,2_/*j*_0,1_ varied among the samples, demonstrating the minimum value for PEG/Cl, which slightly increased after the injection of SPS (Figure 2c). The maximum value was observed at 10 ppm of SPS for the forward scan. For the reverse scan, the ratio *j*_0,2_/*j*_0,1_ similarly had a minimum value for PEG/Cl, while after the injection of SPS, the ratio *j*_0,2_/*j*_0,1_ demonstrated greater variation than it did for the forward scan. Moreover, 15 ppm SPS exhibited a similar value to 10 ppm SPS. This strongly suggests that for the forward scan, the influence of the inner sphere mechanism and ion migration on the total exchange current densities was more balanced than for the forward scan.

On the other hand, Gileadi et al. [38] suggested that the Tafel slope extracted from the CV curves could not serve as an unequivocal tool for building a mechanistic model for establishing the different mechanisms of metal deposition.

Due to the fact of this reason, to gain more insight into the role of SPS in the collapsing of the suppressing abilities of PEG, we examined the surface chemistry using TOF-SIMS spectrometry, and a detailed discussion is provided in the next section.

### 2.2. TOF-SIMS Analysis

#### 2.2.1. Analysis of TOF-SIMS Spectra in the Positive Mode

**Figure 3:**
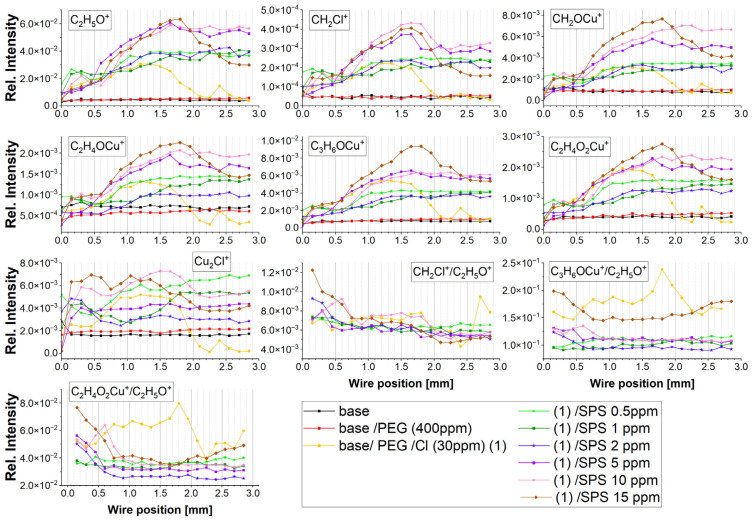
Distribution of intensity of the fragments: C_2_H_5_O^+^, CH_2_Cl^+^, CH_2_OCu^+^, C_2_H_4_OCu+, C_3_H_6_OCu^+^, C_2_H_4_O_2_Cu^+^, Cu_2_Cl^+^, the ratio of CH_2_Cl^+^/C_2_H_5_O^+^, C_3_H_6_OCu^+^/C_2_H_5_O^+^, and C_2_H_4_O_2_Cu^+^/C_2_H_5_O^+^ along the wire position for the base electrolyte, base/PEG, base/PEG/Cl, and base/PEG/Cl/SPS at concentrations of 0.5, 1, 2, 5, 10, and 15 ppm. The wire position corresponds to the applied overpotential during the CV experiment.

The intensity of the distribution of the most characteristic positive fragments identified in the TOF-SIMS spectra as a function of the wire position is shown in Figure 3. The assignments of the identified fragments and their *m*/*z* are listed in Table 1.

The most prominent fragment, C_2_H_5_O^+^, which overwhelmed the rest of the PEG fragments, corresponded to the adsorbed polyethylene glycol fragments. The intensity of C_2_H_5_O^+^ was approximately six times higher than the intensity of the second most prominent fragment, C_3_H_6_OCu^+^. The intensity of the C_2_H_5_O^+^ fragment can be used for estimating the total PEG surface coverage. Furthermore, the intensity of copper adducts with a PEG molecule decreases in the following order: C_3_H_6_OCu^+^, CH_2_OCu^+^, C_2_H_4_O_2_Cu^+^, C_2_H_4_OCu^+^, C_4_H_8_O_2_Cu^+^, and C_3_H_6_O_2_Cu^+^. All the copper–PEG adducts were identified in previous studies [22], and the pathway of the yield of the selected Cu-PEG fragments was proposed. Moreover, the fragment CH_2_Cl^+^, yielded by a secondary reaction of the ^•^CH_2_^•^ diradical with chloride ions during a bombardment of the copper surface during the TOF-SIMS analysis, was identified [22]. Accordingly, the reaction that yields CH_2_Cl^+^ is as follows:

^•^CH_2_^•^ + Cl^•^→ CH_2_Cl^•^ (5)

The proposed mechanism for yielding the fragments C_4_H_8_Cu^+^, C_3_H_6_OCu^+^, CH_2_OCu^+^, C_2_H_4_O_2_Cu^+^, and CH_2_Cl^+^ was provided in the author’s previous paper [22]. However, the suggested pathways do not consider the influence of the PEG conformation on the yield of the individual Cu-PEG fragments. 

Moreover, a more difficult interpretation is related to the Cu-PEG adducts in the presence of MPS molecules adsorbed on the copper surface after the dissociation of SPS. Firstly, it is still unclear whether the copper-PEG adducts correspond to the PEG(Cu^+^) complex formed during electrodeposition or are rather yielded from sputtered copper ions from the copper substrate and PEG molecules during the TOF-SIMS measurements. A previous study [22] proved that Cu-PEG adducts can also be the products of Cu^+^ sputtered ions from the copper surface with the layer of PEG deposited from pure 0.1% ethanolic solution during Bi^+^ bombardment in the TOF-SIMS instrument. However, if we assume that Cu^+^ ions from a Cu^+^(PEG) complex that formed during copper electrodeposition can be entirely dissolved through rinsing, we can assume that the PEG conformation on the Cu/Cl adlayer is preserved. Taking into account this assumption, the TOF-SIMS measurements can be used for the reconstruction of the possible conformation of PEG on the copper surface during copper electrodeposition. Contrarily, if the complex Cu^+^(PEG) did not exist in a parental form during electrodeposition, as it was postulated in [20], the TOF-SIMS experiment can reconstruct the competition of the sulfonate ends of the thiolate molecules with ethereal atoms of PEG in capturing the Cu^+^ ions sputtered from the copper substrate.

Chloride ions in the positive mode of TOF-SIMS were identified in the forms of CH_2_Cl^+^ and Cu_2_Cl^+^. The abundance of the CH_2_Cl^+^ fragment (Reaction 5) is determined by the proximity of Cl^−^ ions to the hydrophobic -CH_2_CH_2_- groups of the PEG skeleton. Under this scenario diradical fragments of ^•^CH_2_^•^ can easily react with Cl^•^, resulting in high yields of CH_2_Cl^•^. Thus, the intensity of the CH_2_Cl^+^ fragment can be used for estimating the number of CH_2_ units attached to the Cl adlayer. A higher yield of CH_2_Cl^+^ can be expected for gauche conformation around C-C, which increases the probability of the closer proximity of a greater number of hydrogens with underlaid chloride adlayer (Figure S6b,c, Supplementary Materials). For trans conformation around C-C (Figure S6a, the dihedral angle along -OCCO- was equal to 180°, which allows only one or two hydrogen atoms to directly contact the chloride adlayer (Figure S6, Supplementary Materials). Decreasing the dihedral angle to 60° (gauche conformation) favors potentially three hydrogen atoms for hydrophobic interaction, while at 0° we can expect the interaction of four hydrogen atoms with the chloride layer. 

The identification of the chloride adlayer was based on the CH_2_Cl^+^ as well as the Cu_2_Cl^+^ fragments. However, CH_2_Cl^+^ unambiguously stands for the chloride ions interacting with PEG, while Cu_2_Cl^+^ can also correspond to the chloride adlayer that is not in proximity to the hydrophobic parts of PEG. 

For the base solution and after the addition of PEG, no characteristic fragments for PEG, SPS, or Cl were detected. Very few chloride ions in the form of Cu_2_Cl^+^ were identified due to the accumulated impurities of chloride on the copper surface [22,62,63], and it was rather constant as a function of the applied overpotential. 

**Figure 4:**
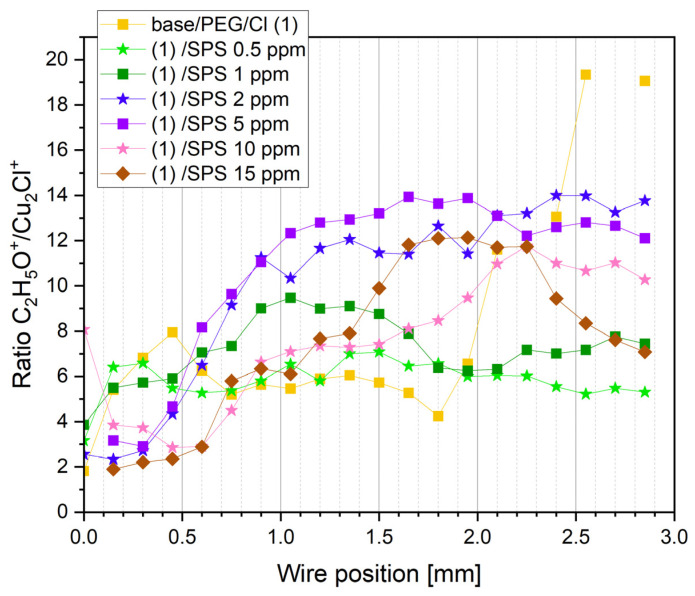
Distribution of the ratio of C_2_H_5_O^+^/Cu_2_Cl^+^ as a function of the wire position for the base solution, base/PEG, base/PEG/Cl, and base/PEG/SPS at concentrations of 0.5, 1, 2, 5, 10, and 15 ppm.

After the injection of chloride ions (30 ppm), characteristic fragments for PEG were identified (sample PEG/Cl, yellow colors). The intensity of the C_2_H_5_O^+^ fragment gradually increased, reaching a maximum of approximately 1.2–1.35 mm, which corresponds to the reverse scan and overpotentials −0.12 and −0.18 V, respectively. A very similar distribution was observed for CH_2_Cl^+^. This strongly suggests that during the increasing adsorption of PEG, when the overpotential was shifting towards the open circuit potential (OCP), the hydrophobic interaction of Cl^−^ ions with hydrogen atoms of the -CH_2_-CH_2_- group occurred. The gauche conformation around C-C was favorable for enhancing and maintaining that effect for a wide range of overpotentials, as it was mentioned above. A roughly similar distribution of the intensity of the Cu-PEG fragments could be observed. Moreover, a good correlation between the distribution of intensity of Cu_2_Cl^+^ and C_2_H_5_O^+^, Cu-PEG adducts, and CH_2_Cl^+^ proves that the Cu_2_Cl^+^ fragment corresponded to the Cl adlayer interacting with PEG. During the reverse scan for the higher overpotentials, the co-desorption of chloride ions (the fragment Cu_2_Cl^+^) and PEG was observed. More detailed information concerning the adsorption/desorption processes can be obtained by examination of the intensity ratio of C_2_H_5_O^+^/Cu_2_Cl^+^ (Figure 4).

For a high overpotential, the ratio of C_2_H_5_O^+^/Cu_2_Cl^+^ increased (positions: 0.0 to 0.45 mm). This was determined by the lower ratio of free chloride ions that did not interact with PEG molecules. Consecutively, for the next examined points (positions: from 0.6 to 0.75 mm), the ratio of C_2_H_5_O^+^/Cu_2_Cl^+^ decreased, which was accompanied by a higher PEG surface coverage. This means that the higher ratio of free chloride ions was not involved in the interaction with PEG. For the positions from 0.75 to 1.65 mm, we can observe a stable value of theC_2_H_5_O^+^/Cu_2_Cl^+^ ratio. This was determined by the fact that a strong synergy of the PEG surface coverage with the chloride ion surface coverage occurred involving a greater number of Cl ions with the interaction with PEG. For a very high overpotentials for the reverse scan, a strong increase in the C_2_H_5_O^+^/Cu_2_Cl^+^ ratio suggests that the chloride layer was desorbed significantly faster than PEG. This observation is supported by the CH_2_Cl^+^/C_2_H_5_O^+^ ratio (Figure 3) in this overpotential region (positions: 1.8–2.0 mm). This phenomenon can be explained by the fact that one molecule of PEG with a molar weight of 8000 consists of approximately 20 monomer units that contain approximately 60 or 80 hydrogen atoms that can interact with the chloride adlayer. Even in a situation where the desorption of half of the chloride ions occurred, the remaining chloride ions could still interact with 10 PEG monomer units, allowing for the remaining PEG molecules on the surface. The injection of 0.5 ppm SPS exhibited only a moderate effect on the C_2_H_5_O^+^/Cu_2_Cl^+^ ratio, while the next portions of SPS (1, 2, and 5 ppm) resulted in an increase in the ratio of the PEG surface coverage to the chloride surface coverage. At the highest concentration of SPS (10 and 15 ppm), the PEG surface coverage, in comparison to the chloride surface coverage, significantly diminished. This behavior was determined by the accumulation of SPS on the chloride adlayer. It is reported that SPS [24,28] is very mobile on the chloride adlayer and after dissociation is chemically bonded through the thiolate end to the copper surface. Under these circumstances, the chloride adlayer is partly displaced by MPS adsorbed [24,63] units and in that way determines the ratio of PEG/Cl. 

The intensity of the Cu-PEG adducts’ distribution was roughly similar (Figure 3). However, it would be interesting to resolve the mutual correlation of the selected Cu-PEG fragments with conformation around C-C and C-O in the PEG skeleton. It seems that the yield of individual Cu-PEG adducts can be determined by the -O-CH_2_-CH_2_-O- conformations, and researchers [19] have distinguished 72 possible conformations of PEG. A recent work [20] suggested that during the shifting potential towards OCP, gauche conformation around C-O is preferred, which allows for adjusting to the optimal distance of 29 Å between the ethereal oxygen atoms to form hydrogen bonds. Moreover, this conformation is optimal for forming a (Cu^+^)PEG complex between two oxygen atoms and Cu^+^ [12,37]. Furthermore, the conformation TGT in the sequence -O-CH_2_-CH_2_-O- is favorable for forming PEG complexes with HgCl_2_ [18] in the crystalline state, while it was suggested that in the liquid state, trans conformation around C-C is more energetically stable. 

Taking into account the above consideration, it is seen that the estimation of the exact conformation around C-O and C-C is challenging, and very often Raman spectra are applied for this purpose. However, the detection limit of normal Raman for PEG is very low. On the other hand, the SERS variant that requires a specially prepared Cu substrate would significantly improve the detection limit. However, a large inspection area is required to receive an optimal Raman signal intensity at a reasonable time of measurement. In our TOF-SIMS analysis, the inspection area was less than 50 µm, which significantly reduces the application of the Raman method. In combination with thiolate, the Raman measurements are additionally more complicated, since many characteristic peaks for MSA overlap with PEG peaks [49].

Due to the above reason, we evaluated Cu-PEG adducts in respect to the possibility of estimating feasible conformations (gauche or trans) around C-C and C-O in PEG. For the examination of the contribution of the conformation around C-O, the intensity of the Cu-PEG adducts (C_3_H_6_OCu^+^ and C_2_H_4_O_2_Cu^+^) was divided by the C_2_H_5_O^+^ intensity, while for determination of the conformation around C-C bonds, the intensity of the CH_2_Cl^+^ fragment was divided by C_2_H_5_O^+^. In this way, the higher ratio of C_3_H_6_OCu^+^/C_2_H_5_O^+^ could correspond to the molecular arrangement, as shown in Figure S6a (Supplementary Materials), while the ratio of C_2_H_4_O_2_Cu^+^/C_2_H_5_O^+^ to the complex structure is depicted in Figure S6b. Likewise, the ratio of CH_2_Cl^+^/C_2_H_5_O^+^ to the molecular arrangement of the hydrophobic groups -CH_2_-CH_2_- in gauche conformation around a C-C bond attached to the Cl adlayer is shown in Figure S6c.

The distribution of the intensity of both the C_3_H_6_OCu^+^/C_2_H_5_O^+^ and C_2_H_4_O_2_Cu^+^/C_2_H_5_O^+^ ratios for PEG/Cl was very similar for the region from 0.0 to 2.0 mm and gradually increased for the forward scan simultaneously with the increasing PEG surface coverage exhibited by the C_2_H_5_O^+^ intensity. For the reverse scan, the C_3_H_6_OCu^+^/C_2_H_5_O^+^ and C_2_H_4_O_2_Cu^+^/C_2_H_5_O^+^ ratios had relatively stable values in the region from 1.0 to 1.5 mm; then, they gave rise to the position from 1.65 to 1.8 mm. For this region, the PEG surface coverage significantly decreased and correlated with the diminished chloride (Cu_2_Cl^+^) surface coverage. This means that during the desorption of PEG at overpotentials of −400 and −490 mV, the contribution of the gauche conformation around C-O (see Table S1, supplement materials) can increase, which is favorable for a higher yield of C_3_H_6_OCu^+^ and C_2_H_4_O_2_Cu^+^. On the other hand, the contribution of the gauche conformation around the C-C bond corresponding to the ratio CH_2_Cl^+^/C_2_H_5_O^+^ was similar at −400 and −480 mV. This means that under the stronger PEG desorption observed at an overpotential of −480 mV, the skeleton of the PEG rotated around the C-C bond due to the desorption of the chloride adlayer, while changes in the C-O rotation were favorable for the stabilization of PEG(Cu+) complexes. The selected conformers of PEG and their influence on the rotation around the C-C bond at the distance of O-O is depicted in Table S1 and Figure S7 (Supplementary Materials). It is clearly seen that rotation around the C-O bond neglected to influence the O-O distance, while rotation around C-C resulted in a dihedral angle O-C-C-O higher than 60°, which significantly increased the O-O length. As a consequence, only under a trans conformation around C-C can the formation of Cu-PEG complexes be ruled out. More detailed quantum chemistry calculations and discussions can be found elsewhere [21]. In this context, the increase in the C_2_H_4_O_2_Cu^+^/C_2_H_5_O^+^ ratio can be primarily determined by the free access to Cu^2+^ ions by ethereal oxygens rather than gauche conformation around C-O. Moreover, when the ratio of CH_2_Cl^+^/C_2_H_5_O^+^ was simultaneously reduced, it was expected to be determined by the partial desorption of the Cl adlayer, which reduced the number of monomer units binding to the Cl ions. However, in this molecular arrangement, PEG molecules were identified on the surface, and ethereal oxygen atoms of PEG can form complexes from the other side of the PEG molecule. 

After the injection of 0.5 ppm SPS, the PEG surface coverage (intensity of C_2_H_5_O^+^) slightly increased. This is in contradiction to the model proposed by other researchers [2,41,42,51], which suggested the partial displacement of PEG by SPS molecules due to the fact of competitive adsorption. The increasing yield of C_2_H_5_O^+^ can be also determined by the existence of MPS molecules on the copper surface due to the matrix effect. However, the higher intensity of all Cu-PEG and CH_2_Cl^+^ rather suggests that synergetic co-adsorption of SPS and PEG occurred under our experimental condition. 

**Figure 5:**
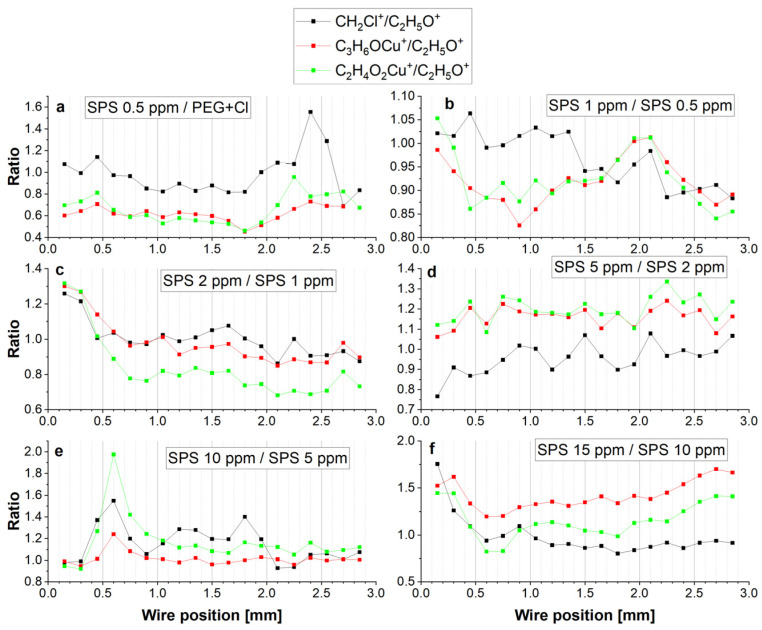
(**a**–**f**). The ratio of intensities of CH_2_Cl/C_2_H_5_O^+^, C_3_H_6_OCu^+^/C_2_H_5_O^+^, and C_2_H_4_O_2_Cu^+^/C_2_H_5_O^+^, obtained by dividing the ratios of the latter fragments of the adjacent sample to the ratio of the former sample. For example, the assignment SPS0.5ppm/PEG/Cl corresponds to the ratio CH_2_Cl^+^/C_2_H_5_O^+^ for SPS 0.5 ppm (adjacent sample) divided by the ratio CH_2_Cl^+^/C_2_H_5_O^+^ for PEG/Cl (the former sample).

For high overpotentials from −600 to −300 mV (positions: 0.0–0.3 mm) for the forward scan as well as for the reverse scan (1.65–2.0 mm), the PEG surface coverage was significantly greater than for the sample without SPS, while for low overpotentials, it was similar. The distribution of C_2_H_5_O^+^ positively correlated with Cu_2_Cl^+^ as well as with the Cu-PEG fragments and CH_2_Cl. However, closer inspection shows a strong reduction, at approximately40%, of the intensity of the C_3_H_6_OCu^+^/C_2_H_5_O^+^ and C_2_H_4_O_2_Cu^+^/C_2_H_5_O^+^ ratios for the sample of PEG/Cl/0.5 ppm SPS in comparison to the sample without SPS (Figure 5a). On the other hand, the CH_2_Cl/C_2_H_5_O ratio only slightly decreased for the reverse scan. This strongly suggests that the introduction of SPS significantly diminished the yields of the Cu-PEG adducts. The first explanation can combine this fact with the changes in the C-O conformation from gauche to trans for a significant number of monomer units of PEG, while the reorientation of the C-C bond from gauche to trans was less severe. However, the yield of C_3_H_6_OCu^+^ seemed to be rather independent of the conformational changes around C-O, since the distance O-O was constant for the different C-O conformations (Table S1, Supplementary Materials), and one ion of Cu^+^ or Cu^2+^ can still easily accommodate in the close proximity of oxygen and form a bond. On the other hand, as it is shown in Figure 5a, the distribution of C_3_H_6_OCu^+^ and C_2_H_4_O_2_Cu^+^ was very similar. This strongly suggests that the primary C_3_H_6_OCu^+^ and C_2_H_4_O_2_Cu^+^ contributions after the addition of SPS was the lack of copper ions in the close proximity of oxygen due to the capture of Cu^2+^ ions by thiolate molecules. Thiolate molecules in gauche conformation are efficient in the partial dehydration of Cu^2+^ and supply Cu^+^ ions directly to the chloride adlayer and in that way omit oxygen atoms of PEG. Moreover, thiolate ions in trans conformations that are less efficient in the latter process can also compete with PEG in capturing Cu^2+^. In that way, the available Cu^2+^ ions for PEG molecules are reduced, diminishing its suppressing role. In this way, the closer chemical environmental surroundings of the ethereal oxygen atom of PEG are diminished in copper ions and metallic copper received after the reduction of Cu^2+^. In consequence, due to the lack of copper atoms in close proximity to the ethereal atom of PEG during the sputtering processes in the TOF-SIMS measurements, the yield of Cu-PEG adducts (i.e., C_3_H_6_OCu^+^ and C_2_H_4_O_2_Cu^+^) were strongly reduced.

The injection of the next portion of SPS (PEG/Cl/SPS1ppm) caused a decrease of both of the C_3_H_6_OCu^+^/C_2_H_5_O^+^ and C_2_H_4_O_2_Cu^+^/C_2_H_5_O^+^ ratios again, similarly as it was observed for PEG/Cl/SPS0.5ppm (Figure 5b), while the ratio CH_2_Cl/C_2_H_5_O was maintained. This means that additional amounts of SPS enhanced the effect of capturing Cu^2+^ ions and, therefore, reduced the number of these ions, so they were not available for PEG.

For greater concentrations of SPS (2 ppm) for higher overpotentials (positions: 0.0–0.6 mm), the PEG surface coverage was reduced (Figure 3), while for the rest of the potential range (positions: 0.75–2.0 mm), it slightly increased in comparison to the PEG/Cl/SPS1ppm. However, the ratio of C_2_H_4_O_2_Cu^+^/C_2_H_5_O^+^ (Figure 5c) was significantly reduced for the range from 0.6 to 2.0 mm and after the CV measurements when switching off of the current occurred (positions: 2.0–2.8 mm). Furthermore, this C_3_H_6_OCu^+^/C_2_H_5_O^+^ ratio was very comparable to that observed for SPS 1 ppm (Figure 3 and Figure 5c). This strongly suggests that at similar surface coverage, expressed by the intensity of the C_2_H_5_O^+^ fragment, the intensity ratio of C_2_H_4_O_2_Cu^+^/C_2_H_5_O^+^ was the main factor that determined the suppressing activities of PEG. This strongly implies that the copper ions that can be captured by two ethereal oxygen atoms (C_2_H_4_O_2_Cu^+^) can form a more stable complex than C_3_H_6_OCu^+^ and, in effect, enhance the suppressing abilities of PEG. In consequence, the lower ratio of C_2_H_4_O_2_Cu^+^/C_2_H_5_O^+^ determines the lower suppressing abilities of PEG. Moreover, the ratio of C_2_H_5_O^+^/Cu_2_Cl^+^ expresses the ratio of the PEG surface coverage to the chloride adlayer surface coverage. Figure 4 depicts that for the samples containing SPS (from 0.5 to 5 ppm), an increase in the PEG surface coverage to chloride surface coverage as a function of the SPS concentration took place, and it demonstrates a similar distribution as the intensity of C_2_H_5_O^+^, while at 10 and 15 ppm of SPS, it was significantly reduced.

The injection of the subsequent portion of SPS (PEG/Cl/SPS5ppm) strongly increased the amount of PEG for low overpotentials for the forward scan (positions: 0.6–1.0 mm) and for the whole reverse scan. Moreover, the ratio of C_2_H_4_O_2_Cu^+^/C_2_H_5_O^+^ increased for the forward as well for the reverse scans in comparison to PEG/Cl/SPS2ppm (Figure 3 and Figure 5d). This means that the suppressing abilities should increase, while the CV curve (Figure 1) demonstrates that the suppressing abilities were still diminished. This inconsistency can be explained by the significantly higher surface coverage of the thiolate observed than that at the SPS concentration. A detailed discussion is provided in the next section.

Similarly, for SPS 10 ppm (Figure 3 and Figure 5e), we can observe the increase in the C_2_H_4_O_2_Cu^+^/C_2_H_5_O^+^ ratio in comparison to SPS 5 ppm. However, the C_2_H_4_O_2_Cu^+^/C_2_H_5_O^+^ and CH_2_Cl^+^/C_2_H_5_O^+^ ratios were comparable for SPS 10 and 15 ppm and demonstrate slightly higher values than for SPS 5 ppm. Moreover, the C_3_H_6_OCu^+^/C_2_H_5_O^+^ ratio was significantly higher for the sample with a greater concentration of SPS (15 ppm). 

#### 2.2.2. Analysis of TOF-SIMS Spectra in the Negative Mode 

The most prominent ions identified in the TOF-SIMS spectra are listed in Table 2.

**Figure 6:**
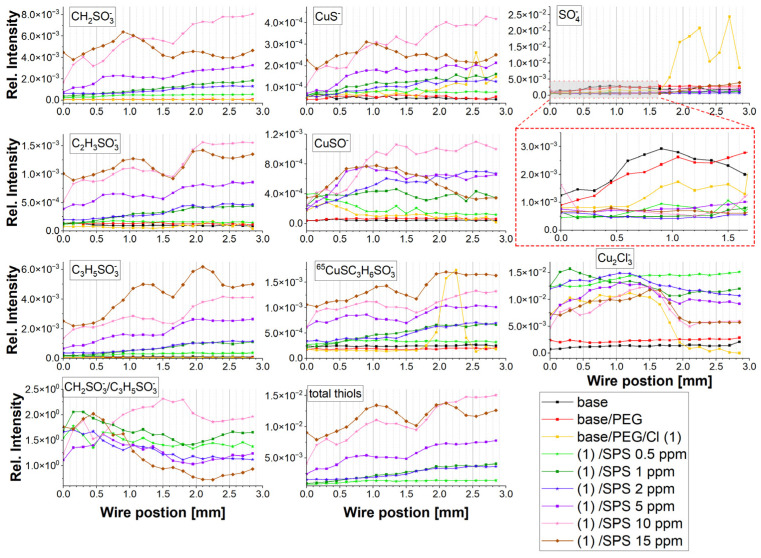
Distribution of the intensity of the fragments: CH_2_SO_3_^−^, CuS^−^, SO_4_^−^, C_2_H_3_SO_3_^−^, CuSO^−^, C_3_H_5_SO_3_^−^, CuSC_3_H_6_SO_3_^−^, Cu_2_Cl_3_^−^, the ratio of CH_2_SO_3_^−^/C_3_H_5_SO_3_^−^, and total thiols along with the wire position for the base electrolyte, base/PEG, base/PEG/Cl, and base/PEG/Cl/SPS at concentrations of 0.5, 1, 2, 5, 10, and 15 ppm. The wire position corresponds to the applied overpotential during the CV experiment.

Figure 6 depicts the intensity distribution of the selected negative fragments: CH_2_SO_3_^−^, CuS^−^, SO_4_^−^, C_2_H_3_SO_3_^−^, CuSO^−^, C_3_H_5_SO_3_^−^, CuSC_3_H_6_SO_3_^−^, Cu_2_Cl_3_^−^, the ratio of CH_2_SO_3_^−^/C_3_H_5_SO_3_^−^, and the total thiols. The total thiols were obtained by summation of the intensities: CH_2_SO_3_^−^, CuS^−^, C_2_H_3_SO_3_^−^, C_3_H_5_SO_3_^−^, and CuSC_3_H_6_SO_3_^−^. 

The sulfate ions were identified in the form of SO_4_^−^ and HSO_4_^−^ while the Cl adlayer was in the form Cu_2_Cl_3_^−^. 

For the base solution, the sulfate ions were adsorbed as the potential increased towards OCP, while after switching to the reverse scan, the sulfate surface coverage gradually decreased (Figure 6). A similar distribution was observed for HSO_4_^−^ (Figure S8, Supplementary Materials). However, the ratio of HSO_4_^−^/SO_4_^−^ demonstrated approximately three times higher values than SO_4_^−^. This ratio is in contradiction to the results reported in [20] that provide evidence suggesting that the SO_4_^−^ ion rather than the HSO_4_^−^ ion dominates on a copper surface immersed in sulfuric acid solution. However, the existence of HSO_4_^−^ ions in the TOF-SIMS mass spectra can be determined by the hydrogen radical ions’ transfer to sulfate ions, which can give rise to the abundance of HSO_4_^−^. This needs a separate study. After the addition of PEG, a minor lower sulfate surface coverage was observed, while after the injection of chloride ions, the significant replacement of sulfate ions by chloride ions was observed. This is consistent with previous studies [63]. The addition of SPS reduced the amounts of sulfate for the whole overpotential range and was roughly similar for all samples at different SPS concentrations. On the other hand, after the addition of SPS (0.5 and 1 ppm), the ratio HSO_4_^−^/SO_4_^−^ was reduced (Figure S8, Supplementary Materials). 

**Figure 7:**
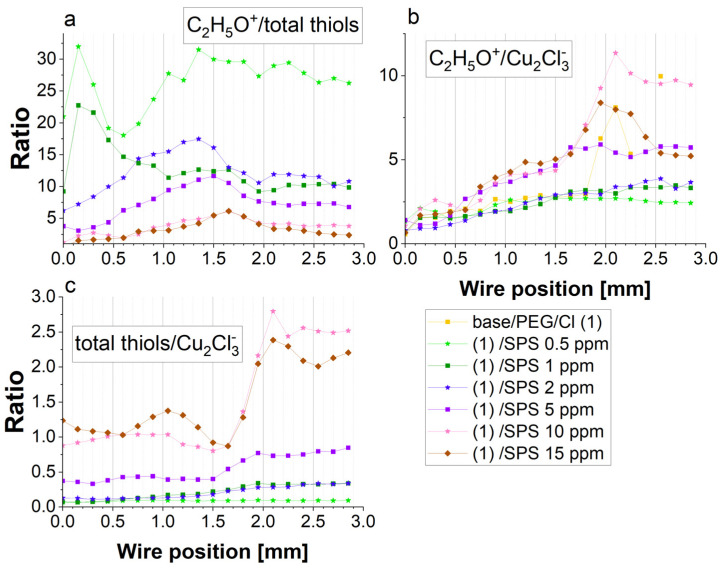
Distribution of the ratio of C_2_H_5_O^+^/total thiols (**a**), C_2_H_5_O^−^/Cu_2_Cl_3_^−^ (**b**), and total thiols/Cu_2_Cl_3_^−^ (**c**) as a function of the wire position for the base solution, base/PEG, base/PEG/Cl, and base/PEG/SPS at concentrations of 0.5, 1, 2, 5, 10, and 15 ppm.

The chloride surface coverage significantly increased after adding 30 ppm Cl ions into the base/PEG solution. The distribution of chloride ions was inversely correlated with chloride ions. This means that chloride ions replaced sulfate ions on the copper surface and vice versa at the region from 1.6 to 2.0 mm, where the strong replacement of chloride ions by sulfate ions was observed. This observation is congruent with previous reports [20,22,63]. After the addition of SPS, the sulfate surface coverage was maintained at the minimum level. This can be determined by the possible impurities adsorbed on the copper surface during a sample’s transfer to the TOF-SIMS instrument. We can assume that sulfate ions are not present on the surface when PEG, Cl, and SPS are adsorbed. The chloride impurities observed for the base and base/PEG solutions were very low and did not demonstrate significant variation as a function of the applied overpotential. After the addition of SPS up to 2 ppm, the Cu_2_Cl_3_^−^ intensity increased, while for the samples with higher concentrations of SPS (5, 10, and 15 ppm), it decreased. This shows that at lower SPS concentrations (up to 2 ppm), chloride ions were co-adsorbed with thiolate ions, while for concentrations of SPS of 5, 10, and 15 ppm, the chloride ions were partly replaced by thiolate ions. The proportion of thiolate/chloride ions is shown in Figure 7. Indeed, the thiolate/chloride ions greatly increased for 5, 10, and 15 ppm of SPS. Accordingly, for 10 and 15 ppm SPS, this ratio was roughly similar, with some visible deviations for 15 ppm, especially for the lower overpotentials for the forward and reverse scans, when the thiolate surface coverage dominated over the chloride surface coverage. 

The distribution of characteristic thiolate ions is shown in Figure 6. More general discussion about the mechanism of yielding both Cu_2_Cl_3_^−^ and thiolate fragments (CH_2_SO_3_^−^, C_3_H_5_SO_3_^−^, and CuSC_3_H_6_SO_3_^−^) can be found elsewhere [22].

A closer inspection of the intensity distributions for the samples with SPS concentrations up to 2 ppm is shown in Figure S9 (Supplementary Materials). 

For the sample PEG/Cl/SPS0.5ppm, we can observe the constant intensity of CH_2_SO_3_ for the positions from 0.0 to 0.5 mm and then the subsequently slight increases for the lower overpotential (0.5–1.0 mm). For the reverse scan, the intensity was constant. Roughly similar behavior can be observed for C_3_H_5_SO_3_^−^. In turn, the CH_2_SO_3_^−^ intensity increased linearly for SPS 1 ppm. While for SPS 2 ppm, for the positions 0.0–0.5 mm, it was unchanged, and for the rest of the overpotentials (forward and reverse scan), it increased linearly, demonstrating similar values to that of SPS 1 ppm. A significantly higher thiolate surface coverage was observed at 5, 10, and 15 ppm of SPS. The intensity of the fragment C_3_H_5_SO_3_^−^ for 0.5, 1, 2, and 5 ppm of SPS in comparison to CH_2_SO_3_^−^ slightly increased in the range of 0.65 to 2.0 mm (Figure 6), since the ratio CH_2_SO_3_^−^/C_3_H_5_SO_3_^−^ slightly decreased. Recently [63], we showed that the ratio of CH_2_SO_3_^−^/C_3_H_5_SO_3_^−^ can be assigned to the gauche/trans conformation of MPS molecules adsorbed on the copper surface. On the other hand, at 10 and 15 ppm SPS, we could observe a great variation in the ratio of CH_2_SO_3_^−^/C_3_H_5_SO_3_^−^. For 10 ppm, in the range of 0.64 to 1.5 mm, the CH_2_SO_3_^−^/C_3_H_5_SO_3_^−^ ratio strongly increased, while at 15 ppm of SPS, it strongly decreased. This previously suggests [37,63] that the gauche conformation of thiolate molecules is favorable to the partial dehydration of Cu^2+^ ions and after the reduction transfer of Cu^+^ ion to the chloride adlayer. In terms of functionality, we can expect significantly higher accelerating abilities for 10 ppm than for 15 ppm for the reverse scan. This is consistent with the CV curves for 10 and 15 ppm of SPS (see Figure 1). 

The scale of acceleration should be estimated based on the total thiolate surface coverage (Figure 6), the gauche/trans ratio (Figure 6), the thiolate/chloride ratio (Figure 7c), and the ratio of PEG/thiolate expressed as C_2_H_5_O^−^/thiolate (Figure 7a) as well as PEG/chloride expressed by C_2_H_5_O^−^/Cu_2_Cl_3_^−^ (Figure 7b). As it is depicted in Figure 6, the thiolate surface coverage, calculated as the sum of the intensity of CH_2_SO_3_^−^, C_2_H_3_SO_3_^−^, C_3_H_5_SO_3_^−^, and CuSC_3_H_6_SO_3_^−^, was slightly higher at 15 ppm than at 10 ppm of SPS. However, as was shown above, significantly higher thiolate molecules remained in the trans conformation for 15 ppm of SPS. Even though the total number of MPS molecules in the gauche conformation was still slightly greater at 10 ppm than for 15 ppm for the forward scan, a greater contribution of the trans conformation (C_3_H_5_SO_3_^−^) may decrease the accelerating effect of thiolate in the gauche conformation for PEG/Cl/SPS15ppm. 

For 5 ppm SPS, the thiolate total surface coverage was moderate, while for 0.5, 1, and 2 ppm, it was significantly lower. An interesting situation was observed at 1 and 2 ppm, since we observed a very similar distribution of thiolate surface coverage and a slightly lower CH_2_SO_3_^−^/C_3_H_5_SO_3_^−^ ratio for 2 ppm SPS. Since the CV curves showed that for 2 ppm, the accelerating effect was stronger than for 1 ppm, the results based on the negative ions cannot supply us with the relevant answer for why that effect was not significantly observed in the distribution of the negative ions. Additional insight can be received when we compare the distribution of the thiolate surface coverage with the PEG surface coverage. 

Figure 7a depicts the distribution of the C_2_H_5_O^+^/thiolate ratio. For 1 ppm SPS, the C_2_H_5_O^+^/thiolate ratio decreased for the forward scan, while for 2 ppm, the C_2_H_5_O^+^/thiolate ratio increased. The crossing point was observed at 0.75 mm, while for low overpotentials for the forward scan for 2 ppm SPS, the relative PEG surface coverage in comparison to thiolate was slightly greater than for 1 ppm. The latter fact suggests that the suppressing abilities can slightly dominate for 2 ppm SPS over 1 ppm SPS for the reverse scan, which is in contradiction with the CV curve (Figure 1). It seems that the resultant electrochemical effect was determined not only by the ratio of C_2_H_5_O^+^/total thiols through the effective capturing of Cu^2+^. More precisely, the latter effect could be related to the interactions with hydrated Cu^2+^ ions, as it was supposed by Liu et al. [20]. It was reported [37] that sulfonate ions in the gauche conformation can efficiently increase the velocity of the partial dehydration of Cu^2+^. On the contrary, it is likely that PEG molecules can interact with hydrated Cu^2+^ by two ethereal oxygen atoms due to the appropriate fit of the O-O distance (2.9 Å for a dihedral angle -O-C-C-O- at 60 degrees; see Table S1 and Figure S7, Supplementary Materials) in PEG and the O-O distance in hydrated water of Cu^2+^. After capturing the Cu^2+^ by MPS units by ion–pair interaction, the Cu^2+^ ion was not available for PEG and vice versa. For the examination of the Cu^2+^ ions capturing scale by PEG molecules, the C_2_H_4_O_2_Cu^+^/C_2_H_5_O^+^ ratio can be used (Figure 3). It was clearly seen that for 2 ppm of SPS, the C_2_H_4_O_2_Cu^+^/C_2_H_5_O^+^ ratio strongly decreased in the range of 0.0–0.75 mm and demonstrated a significantly lower value than for 1 ppm SPS (Figure 5c) for the region 0.6–2.0 mm and under OCP after the CV experiment (positions: 2.0–3.0 mm). The latter fact can explain the stronger accelerating abilities for 2 ppm SPS, since the accelerating role of thiolate contribution was not compensated for by the suppressing abilities of PEG. Thus, the resultant electrochemical abilities were determined by the influence of the suppressing properties of PEG based on the interaction of ethereal oxygen atoms of PEG with hydrated Cu^2+^ and the accelerating abilities of the MPS that was connected with the gauche conformation of MPS, favored for trapping Cu^2+^. The latter hypothesis was proposed by spectroscopic studies on the electrochemistry of PEG/Cl [20], while the role of the gauche conformation of thiolate molecules was examined by SHINERS elsewhere [37]. On the other hand, similar spectroscopic studies devoted to the electrochemical properties of PEG/SPS/Cl were not provided. Here, we have to point out that the TOF-SIMS data do not supply information regarding possible changes in the “structural water” observed at the interface of Cu/Cl/PEG, which were considered and examined by Liu et al. [20] and Schmitt et al. [37]. 

An analogous interpretation can be provided for the samples of PEG/Cl/SPS10ppm and PEG/Cl/SPS15ppm. For the forward scan, the CV curves were very similar for 10 and 15 ppm of SPS, while for the reverse scan, a significant increase in the accelerating abilities was observed. For 10 ppm SPS, at the region from 0.0 to 0.6 mm, the contribution of the gauche conformation around C-C (CH_2_Cl^+^/C_2_H_5_O^+^) and the C_2_H_4_O_2_Cu^+^/C_2_H_5_O^+^ ratio increased, while for the low overpotentials, less than −0.25 V, it significantly decreased, which was accompanied by a significant increase in the PEG surface coverage for that region. For the reverse scan, the contribution of the gauche conformations about C-C and the C_2_H_4_O_2_Cu^+^/C_2_H_5_O^+^ ratio was rather constant. Different behavior was observed at 15 ppm SPS, when a significant decrease in the gauche conformation was observed for the higher overpotentials (positions: 0.0–0.75 mm) and, subsequently, remained rather constant for the rest of the CV curve. However, for the reverse scan, the contribution of the C_2_H_4_O_2_Cu^+^/C_2_H_5_O^+^ ratio for 15 ppm SPS was higher than at 10 ppm of SPS, while the gauche conformation about C-C was slightly more dominant at 10 ppm SPS than for 15 ppm SPS. 

Moreover, for these samples (10 and 15 ppm SPS), both the total PEG surface coverage (C_2_H_5_O^+^) as well as the ratio of C_2_H_5_O^+^/thiolate was very similar. However, we can observe a significant increase in the ratio of C_3_H_6_OCu^+^/C_2_H_5_O^+^. The fragment of C_3_H_6_OCu^+^ corresponded to a stable complex, while for the forward scan, we did not see any differences between 10 and 15 ppm SPS. Due to the fact of this reason, the conformation that was favorable for a higher yield of C_3_H_6_OCu did not have a major impact on the potentially greater suppressing properties of PEG. Careful inspection of the CH_2_SO_3_^−^ fragment clearly shows that the switching of the CV scan from forward to reverse determined the changes in the gauche conformation for 15 ppm SPS, while this effect was not observed for 10 ppm. For the forward scan, the gauche conformation increased, while for the reverse scan, it diminished. As a consequence, the ratio of the gauche/trans of the thiolate molecules strongly dominated for the reverse scan for 10 ppm over 15 ppm SPS (Figure 6), determining the higher accelerating abilities. 

Interestingly, the variation in the CH_2_SO_3_^−^/C_3_H_5_SO_3_^−^ ratio at 15 ppm SPS correlated quite well with the surface coverage of PEG. The reduction of the PEG surface coverage observed around position 0.5 mm (overpotential: −0.3 V) and the subsequent increase determined the significant rotation of the PEG skeleton around the C-C and C-O bonds. Moreover, the strong correlation of CH_2_Cl^+^ with both Cu_2_Cl and Cu_2_Cl_3_^−^ for the entire range of wire strongly suggests the hydrophobic interaction of the CH_2_-CH_2_ group of PEG with the Cl adlayer, while the rotations around C-O were relatively independent of the Cl adlayer. Interestingly, for very high overpotentials for the reverse scan, the amounts of Cu-PEG were still maintained, while the hydrophobic interaction diminished due to the reduction of the Cl surface coverage. This means that under a high thiolate surface coverage (15 ppm), the PEG can remain on the surface even if the hydrophobic interaction of the CH_2_CH_2_ groups was significantly reduced due to the decreasing availability of Cl sites on the copper. This suggests that ethereal atoms of PEG can efficiently interact with the copper surface when PEG molecules are stabilized by the gauche conformation of thiolate molecules under a desorption regime. 

However, the primary reason for the conformal reorientation of both PEG and thiolate was the higher accumulation of PEG and thiolate monolayers on the copper surface. As a consequence, we could observe higher numbers of Cu-PEG adducts at 15 ppm SPS than for 10 ppm SPS, while CH_2_Cl slightly diminished. Moreover, significantly higher thiolate surface coverage in the trans conformation was responsible for reducing the accelerating abilities.

The CuS^−^ fragment corresponded to the thiolate ends of the MPS molecules after dissociative adsorption of SPS on the copper surface. A careful inspection of Figure 4 shows that Cu-S positively correlated with the CH_2_SO_3_^−^ fragment, while C_3_H_5_SO_3_^−^ demonstrated a slightly different distribution. On the other hand, the distribution of CuSC_3_H_6_SO_3_^−^ was more positively correlated with the C_3_H_5_SO_3_^−^ fragment than with CH_2_SO_3_^−^. These differences can be determined by the fact that thiolate molecules in the gauche conformation (CH_2_SO_3_^−^) can be positioned more perpendicular to the Bi primary during the TOF-SIMS measurements, and in this way, the yield of CuSC_3_H_6_SO_3_^−^ is reduced for this conformation due to the higher fragmentation of molecule. On the contrary, under the trans conformation, MPS molecules are tilted more parallel to the primary Bi beam, preserving the MPS molecules bonded to the copper surface against higher fragmentation. As a consequence, a higher yield of CuSC_3_H_6_SO_3_^−^ was observed. At this point, it should be expressed that the primary Bi beam strikes the sample at an angle of 45°.

The assignment of the CH_2_SO_3_^−^ to the gauche and C_3_H_5_SO_3_^−^ to the trans conformation was recently reported [63].

For the sample PEG/Cl/SPS2ppm, we can observe a reduced ratio of CH_2_SO_3_^−^/C_3_H_5_SO_3_^−^ that corresponded to the gauche/trans ratio. The decreased value of the gauche conformer (CH_2_SO_3_^−^) was accompanied by a reduced intensity of Cu-S. 

This hypothesis is supported by the intensity distribution of the CuSO^−^ fragment. The latter fragment can be assigned to the sulfonate ends and is formed by the reaction of the SO_3_ group with sputtered copper during Bi bombardment in the TOF-SIMS instrument. The distribution of CuS^−^ demonstrated generally good agreement with CuS^−^ while exhibiting more distinct differences between samples. However, some inconsistency was observed for 5 and 15 ppm SPS, when a very similar distribution of CuSO^−^ was observed, while for CuS^−^ it was significantly different. 

**Figure 8:**
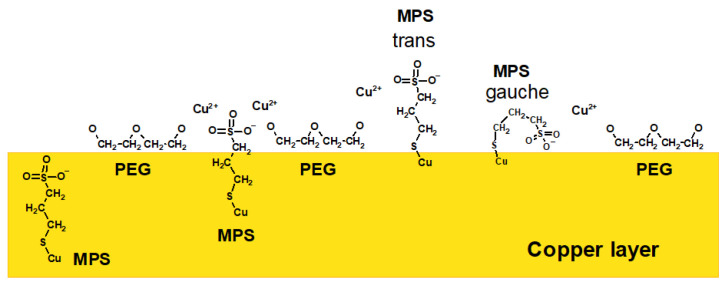
Possible molecular arrangements of MPS and PEG molecules on and into the copper layer during electrodeposition. Please note that some MPS molecules are incorporated into the copper deposit. The chloride adlayer was omitted for clarity.

Overall, the interaction between PEG and SPS on the copper surface under reactive conditions (copper electrodeposition) is illustrated in Figure 8. The chloride adlayer was omitted for clarity. SPS molecules after dissociative adsorption on the copper surfaces reduce into two MPS units, as postulated by Hai et al. and proven by other investigations [22,23,28,66]. However, the model postulated by Hai et al. does not take into account the incorporation of thiolate molecules into the copper deposit. The latter fact was proved recently [63] and suggested previously [50,67]. On the contrary, PEG molecules are not able to incorporate into a copper deposit, as suggested previously [22,50]. PEG molecules are bonded to the chloride adlayer through the hydrophobic -CH_2_CH_2_- part of PEG. This fact was suggested previously for PEG/Cl [20,21,22], and it was proven in that work for the PEG/SPS/Cl component of an electroplating bath. Contrary to previous studies, PEG was co-adsorbed with SPS under our experimental conditions in an SPS concentration range from 0.5 to 15 ppm. However, the ratio of MPS/PEG increased as a function of the SPS concentration. The first function of MPS in decreasing the PEG-suppressing role relies on the limited access of hydrophobic parts to the chloride adlayer due to the partial displacement of chloride ions by MPS molecules. In this way, ethereal oxygen atoms are pointed out toward the solution at a greater distance from the electrode surface. This latter fact minimized their effectiveness in capturing Cu^2+^. On the other hand, MPS molecules are very efficient at capturing Cu^2+^, especially in gauche conformations, as is schematically shown in Figure 8. The formation of Cu-MPS bonds can be found in our previous paper (Figure 11) [63]. The main idea depicted in Figure 8 is the demonstration that PEG exists only on the copper surface, while MPS molecules can also be incorporated into the copper deposit and exhibit competition in catching Cu^2+^ ions.

**Figure 9:**
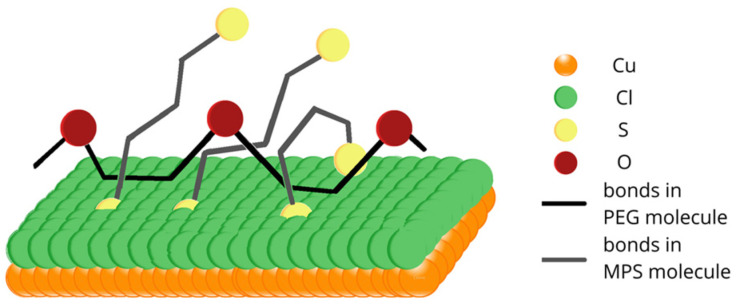
3D projection of the possible molecular arrangements of MPS adsorbed units in a PEG molecular surrounding.

One of the possible molecular arrangements of MPS thiolate molecules and PEG in 3D projection is shown in Figure 9. All hydrogen atoms and oxygen atoms located around sulfur pointed towards the top were omitted for clarity. MPS molecules (sulfur atoms indicated by the yellow color and bonds by grey) penetrate and disrupt the chloride adlayer by forming chemical bonds with the copper substrate. On the contrary, PEG molecules (oxygen atoms in PEG are indicated by the brown color and bonds by black) can interact with the chloride adlayer through hydrophobic interactions. Due to the high mobility of MPS and the high length of PEG 8000, MPS and PEG molecules can be wrapped around each other. In this model, sulfonate groups efficiently capture Cu^2+^ ions and, in this way, reduce similar abilities for the ethereal oxygen of PEG. The proposed mechanism is in contradiction to the model postulated by Hai et al. [23], where the primary antisuppressing effect of MPS relies on the partial rupturing of coordination bonds of Cu(+)-Cl- in the PEG(Cu^+^)Cl^−^ complexes by displacement of Cl ions from the copper surface. The second antisuppressing effect of MPS molecules is connected with the coordinative dissolution of the PEG-(Cu+)Cl- complex by desorbed MPS units. However, the existence of PEG(Cu+)Cl- complexes that play a central role in the suppressing abilities of PEG was undermined recently [20].

## 3. Materials and Methods

The base electrolyte contained sulfuric acid (99.9% pure, POCH S.A., Gliwice, Poland) and copper sulfate (99% pure, Supelco). The following additives were added: Cl^−^ (in the form of HCl, POCH S.A., Gliwice, Poland), SPS (Raschig, Ludwigshafen, Germany), and polyethylene glycol (molecular weight: 8000; Sigma-Aldrich, Saint Louis, MO, USA). For the preparation of all solutions, deionized water (18 MΩ, Hydrolab, Straszyn, Poland) was applied. For the cyclic voltammetry experiment (CV), a 120 mm piece of nitinol wire (0.665 mm in diameter, supplied by Euroflex GmbH, Pforzheim, Germany) was immersed into a copper sulfate solution in the length of 50 mm (1 cm^2^ surface area).

The cyclic voltammetry experiment (CV) was accompanied by TOF-SIMS experiments, where a copper ring with phosphor as an additive (diameter: 45 mm outside and 30 mm inside; thickness: 1 mm; supplied by Lesker, Jefferson Hills, PA, USA) was employed as a counter electrode and mounted on the bottom of the test tube. The tube contained 250 mL of electroplating bath. The purity and manufacturer of the components of the electroplating bath are mentioned above, while the concentration of the base solution (without additives) was as follows: CuSO_4_ × 5H_2_O—0.225 M and sulfuric acid—0.56 M. Accordingly, PEG (400 ppm), Cl^−^ ions (30 ppm), and SPS (0.5, 1, 2, 5, 10, and 15 ppm) were added as additives.

In cyclic voltammetry experiments (CV) accompanied by TOF-SIMS experiments, a copper ring with phosphor as an additive (45 mm outside and 30 mm internal diameter; 1 mm in thickness; supplied by Lesker, Jefferson Hills, PA, USA) was employed as a counter electrode and mounted on the bottom of the test tube. A counter electrode was connected to a galvanostat by a copper wire (diameter 1 mm) covered by a thick isolated lacquer layer that was resisted for the electroplating bath. As a reference, a sulfate electrode, Ag/Ag_2_SO_4_, was applied (supplied by EuroSensor, Gliwice, Poland). A galvanostat AUTOLAB 302N (Eco Chemie, Utrecht, The Netherlands) was used for the cyclic voltammetry (CV) experiments. Before the addition of the sulfuric acid and additives, a copper sulfate solution was thoroughly deoxygenated using an ultrasonic washer under reduced pressure. Before each experiment, the counter electrode was immersed into fresh 30% HNO_3_ for 30 s, then thoroughly rinsed in deionized water and dried in the air. Subsequently, nitinol wire, which served as a working electrode, was mounted vertically in the electroplating bath, and copper was electrodeposited at a current density of −20 mA/cm^2^ for 30 s. Subsequently, the open circuit potential (OCP) was determined for 30 s or less before the CV experiment until a steady state was reached. After the OCP determination preplated nitinol wire was withdrawn at a constant speed equal to 34 µm/s from the copper electroplating bath under electrodeposition conditions. Simultaneously, the CV experiment was performed, and the applied potential scan speed was swept at a constant value, V = 20 mV/s, in the range of overpotential between −0.6 V and OCP (forward scan to anodic direction) and from OCP to −0.6 V (reverse scan to cathodic direction), where OCP is the open circuit potential. In this manner, during the CV experiment, one cyclic loop corresponded to 60 s (30 s for the forward scan in the anodic direction and 30 s for the reverse scan in the cathodic direction) and 2 mm of the nitinol wire (working electrode) length during a withdrawal. A forward scan from −0.6 V to OCP corresponded to the wire positions from 0 to 1 mm, while the reverse scan corresponded to the positions from 1 to 2 mm. The velocity of 34 µm/s for the wire withdrawing from the solution was matched with a CV scan rate of 20 mV/s. In this way, the first 1 mm of wire that was withdrawn over 30 s corresponded to the CV forward scan, while the second 1 mm of the wire (positions: 1 to 2 mm) was matched to the reverse scan, as is shown in Figure 1c. This allowed us to assign the position of the wire to the applied potential and could be easily localized in subsequent TOF-SIMS measurements. Exactly the same procedure was applied in our two previous works [22,63]. The electroplating setup can be found elsewhere (Figure 18, in [22]). The scheme of the experiment combining the CV experiment with the wire withdrawal can be found as a graphical abstract in our previous paper [63]. 

To avoid copper sulfate crystallization on the copper surface after the CV experiment, the optimized method of rinsing was applied. More details on the rinsing method can be found elsewhere [22]. Briefly, a glass capillary with an internal diameter of 2.4 mm was immersed into a rinsing solution (5% ethanol) and mounted horizontally. The total volume of the rinsing solution inside the capillary was equal to 8 µL (height of the rinsing liquid: ~1 mm) and was collected at the bottom end of the capillary. The rinsing properties of 5% ethanol in deionized water and other concentration of ethanolic solutions were determined in the previous work [22]. The optimal results were received for 5% ethanolic solution that, from one side, effectively dissolved the remaining CuSO_4_ and, from the other side, preserved the maximum amount of PEG and thiolate on the copper surface. However, since thiolate is incorporated into the copper deposit [22], the risk of removing thiolate molecules does not occur. Subsequently, a sample in a vertical position was withdrawn at a constant speed of V = 800 µm/s through the rinsing liquid and then immediately dried in air. As the inspected length of the sample was routinely no longer than 4 mm, the volume of the rinsing liquid was sufficient for cleaning the copper sulfate that remained. On the other hand, the reduced volume of the rinsing liquid and the drying step reduced the contact time of the liquid with the underlying PEG/MPS/SPS film, decreasing the risk of solubility of the organic film.

After preparation, the samples were transferred to the TOF-SIMS vacuum chamber and measured routinely no later than 2–5 h after preparation. The TOF-SIMS spectra were acquired by means of the TOF-SIMS.5 instrument (ION-TOF GmbH, Münster, Germany). The primary ion source of Bi^+^ was used at 30 keV (cyclic time: 100 µs; primary beam current: 1.2 pA). The scanning area of the secondary ions was 50 × 50 µm with 128 × 128 pixels and 1 shot/pixel. All the measurements were performed under the static mode (dose no larger than 1 × 10^12^ ions/cm^2^) in a negative mode. For most of the samples, 30 or more points separated by a 0.1 mm distance were measured. The post-processing data analysis was conducted using SurfaceLab 6.7 software (ION-TOF) and Origin 2019 (OriginLab, Northampton, MA, USA). The negative spectra were recorded and calibrated using the positions of CH^−^, CH_2_^−^, and CH_3_^−^, while the positive spectra used positions of CH_2_^+^, C_2_H_3_^+^, C_3_H_5_^+^. The intensities were normalized to the total intensity.

## 4. Conclusions

In this work, we examined, for the first time, the interactions between polyethylene glycol (PEG) and SPS in the presence of a chloride adlayer by TOF-SIMS combined with cyclic voltammetry. The received results show that SPS after dissociative adsorption on the copper surface in the state of MPS significantly reduced the inhibiting properties of PEG. The enhancing accelerating abilities were proportional to the concentration of SPS in the range from 0.5 to 15 ppm. The effect of the acceleration was not symmetrical for both the forward and reverse scans. 

We identified in the TOF-SIMS spectra characteristic Cu-PEG adducts in the positive mode and characteristic thiolate fragments in the negative mode. The identification of CH_2_Cl^+^ fragments proves that the hydrophobic interactions of PEG rely on the close proximity of CH_2_-Cl due to the favorable gauche conformation about the C-C bonds. Moreover, the identification of C_2_H_4_O_2_Cu^+^ and C_3_H_6_OCu^+^ adducts determined the possibilities of forming PEG-Cu(+) complexes and their suppressing abilities. The accelerating abilities of SPS were determined by both the thiolate surface coverage and the ratio of the gauche/trans conformation.

Firstly, the TOF-SIMS data showed that the addition of SPS led to co-adsorption of SPS and PEG, while the ratio of the PEG surface coverage to the thiolate surface coverage decreased as a function of the SPS concentration. However, for 0.5 ppm SPS, a significant deviation from the distribution of PEG/thiolate was observed. Moreover, the PEG/thiolate ratio was similar for 10 and 15 ppm SPS. Secondly, a strong reduction in the number of both C_2_H_4_O_2_Cu^+^ and C_3_H_6_OCu^+^ adducts was observed after the SPS injection. This means that the thiolate molecules were able to efficiently capture Cu^2+^ ions from the ethereal oxygen surroundings of PEG. Since we did not observe a proportional reduction in CH_2_Cl^+^, it means that the gauche conformation of PEG around C-C was significantly preserved. Based on that observation, we can conclude that the distance of the O-O ethereal atoms was also preserved. Due to the fact of this reason, we can conclude that competition between SPS and PEG in capturing Cu^2+^ ions relies on the higher affinity and flexibility of the sulfonate groups of thiolate molecules.

## Figures and Tables

**Table 1:** The assignment and *m*/*z* (mass to charge) of the most distinct positive fragments identified in the TOF-SIMS mass spectra.

*m*/*z*	Assignment
45.00	C_2_H_5_O^+^
48.91	CH_2_Cl^+^
92.79	CH_2_OCu^+^
106.78	C_2_H_4_OCu^+^
120.77	C_3_H_6_OCu^+^
122.75	C_2_H_4_O_2_Cu^+^
150.72	C_4_H_8_O_2_Cu^+^
160.55	Cu_2_Cl^+^

**Table 2:** The assignment of the most distinct negative fragments identified in the TOF-SIMS mass spectra.

No.	Centre Mass (u)	Assignment
1	94.02	CH_2_SO_3_^−^
2	94.95	CuS^−^
3	96.00	SO_4_^−^
4	97.00	HSO_4_^−^
5	107.03	C_2_H_3_SO_3_^−^
6	110.95	CuSO^−^
7	121.05	C_3_H_5_SO_3_^−^
8	219.01	^65^CuS(CH_2_)_3_SO_3_^−^
9	230.87	Cu_2_Cl_3_^−^

## Data Availability

The TOF-SIMS data can be obtained upon reasonable request.

## Supplementary Materials

The following supporting information can be downloaded at: https://www.mdpi.com/article/10.3390/molecules28010433/s1, Figure S1: Distribution of OCP determined on the preplated copper layer directly before CV experiments. OCP was determined by means of sulfate Ag/Ag_2_SO_4_ reference electrode to reach a steady state value. The time needed to obtain the steady state value is shown for comparison; Figure S2: The linear regression applied for the determination exchange current density *j*_0,1_ for a base electrolyte for forward scan for overpotentials lower than 20 mV.; Figure S3: The linear regression applied for the determination of exchange current density *j*_0,2_ for a base electrolyte for forward and reverse scan for overpotentials from −150 mV to −200 mV. Figure S4: Distribution exchange current density *j*_0,2_ as a function of SPS injected to the copper electroplating solution containing PEG/Cl.; Figure S5: Distribution exchange current density *j*_0,1_ as a function of SPS injected to the copper electroplating solution containing PEG/Cl; Figure S6: The proposed, simplified molecular model of C_3_H_6_OCu^+^ (a) and C_2_H_4_O_2_Cu^+^ (b) fragments and hydrophobic interaction of PEG with chloride adlayer (c).; Figure S7: The O-O distance as a function of dihedral angle -OCCO- for monomer unit of PEG.; Figure S8: Distribution of intensity hydrosulfate and sulfate ions along the wire position for: base solution, base+PEG, base/PEG/Cl and base/PEG/Cl/SPS0.5 ppm; Figure S9: Intensity distribution of negative fragments identified in the TOF-SIMS mass spectra for samples containing SPS at concentration up to 2 ppm.; Table S1: The selected 3D projections for monomeric unit of PEG. Projections 1–5 corresponds to the molecular models rotated round C-C bond at the fixed dihedral angle C-C-O-C 0°. Projection 6 depicts PEG(Cu+) complex. Projections 7–11 corresponds to the conformers 1–5 while at dihedral angle 60°.

## Sample Availability

Samples of the compounds are not available from the authors.
