# Supplementary material for: Interaction of Bis-(sodium-sulfopropyl)-Disulfide and Polyethylene Glycol on the Copper Electrodeposited Layer by Time-of-Flight Secondary-Ion Mass Spectrometry
Source: Molecules. 2023 Jan 3;28(1):433. doi: 10.3390/molecules28010433 (PMC9824609; doi:10.3390/molecules28010433)
Supplement: Supplementary file 1 [file molecules-28-00433-s001.zip › molecules-2105096-supplementary.pdf]

Table S1. The selected 3D projections for monomeric unit of PEG. Projections 1-5 corresponds to the molecular models rotated round C-C bond at the fixed dihedral angle C-C-O-C 0° . Projection 6 depicts PEG(Cu+) complex. Projections 7-11 corresponds to the conformers 1-5 while at dihedral angle 60°

Table S1.

Dihedral angle O-C-C-O	O-O distance [Å]	3D projection	Dihedral angle C-C-O-C	O-O distance [Å]	3D projection
0°	2.55	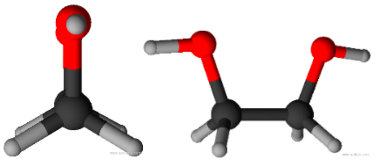	60°	2.55	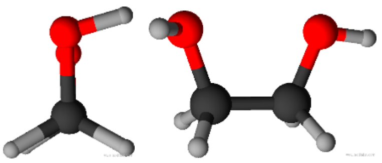
30°	2.642	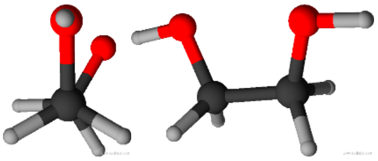		2.642	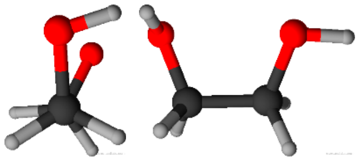
60°	2.879	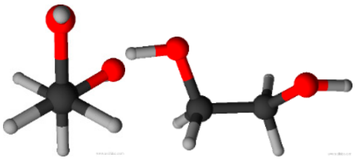		2.879	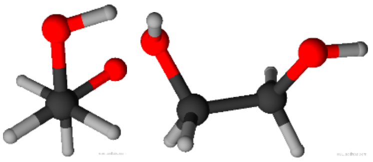
90°	3.174	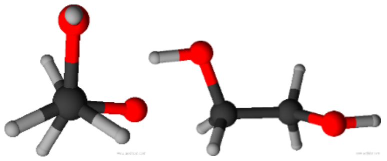		3.174	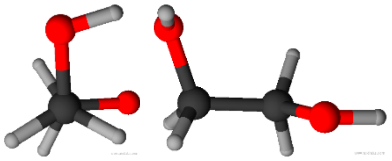
180°	3.669	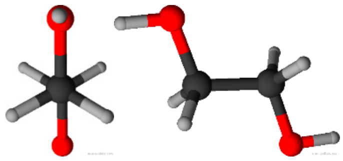		3.669	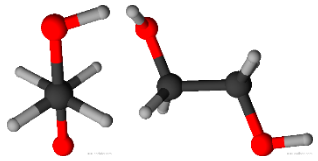
0.645	2.781	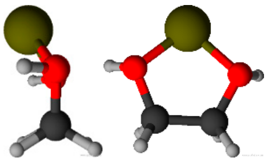			

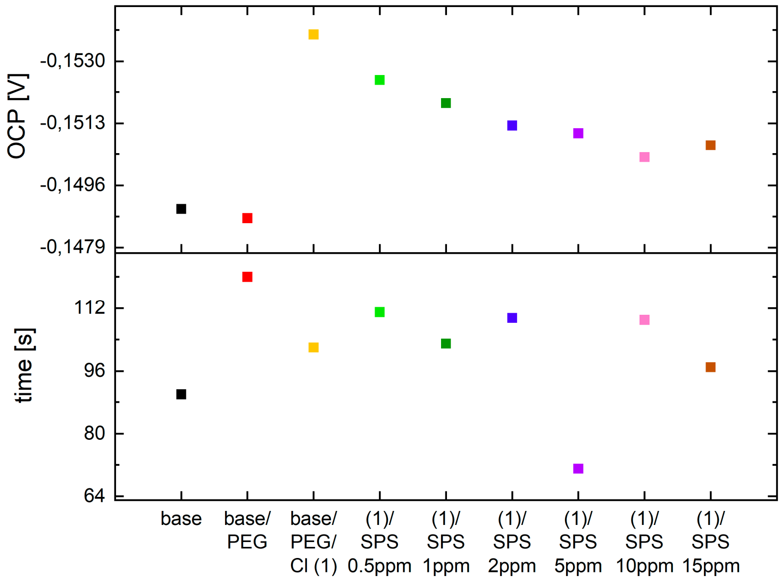

Figure S1 Distribution of OCP determined on the preplated copper layer directly before CV experiments. OCP was determined by means of sulfate Ag/Ag_2SO_4 reference electrode to reach a steady state value. The time needed to obtain the steady state value is shown for comparison.

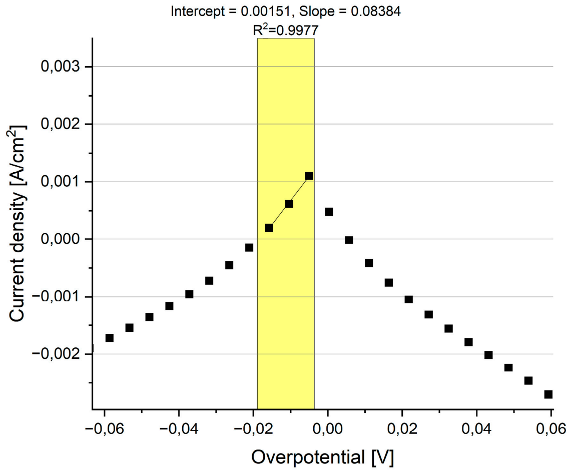

Figure S2 The linear regression applied for the determination exchange current density $j_{0,1}$ for a base electrolyte for forward scan for overpotentials lower than 20 mV.

The data shown in Figure S7.1 was fitted to simplified version of the Butler-Volmer equation (1) that can be limited to the first term of the Taylor series for very low overpotentials, similarly as it was applied by other researchers [37] and it was proposed by classical electrochemical book [64]

$$j = j_{0,1}\left(\frac{nF\eta}{RT}\right) = j_{0,1}\left(\frac{\eta}{0.0257}\right) \quad (1)$$

where: $j_{0,1}$ denotes the exchange current density for the Cu^+ to Cu reduction, n is the number of transferred electrons (n = 1), F – Faraday constant (C/mol), η – the overpotential (V), R – gas constant (J/(mol*K)), T – temperature (K).
After putting R and F constants into the equation (1) for T=298 K, n=1 the equation (1) is as follows

$$j = j_{0,1} * 0.039\ \eta, \tag{2}$$

The Tafel slope value corresponded to $j_{\ 0,1} * 0.039$ was calculated by linear regression (slope = 0.08384) as it is shown in Figure S6.1 In that way $j_{0,1} * 0.039 = 0.08384$ and $j_{0,1} = 2.15\ mA$.

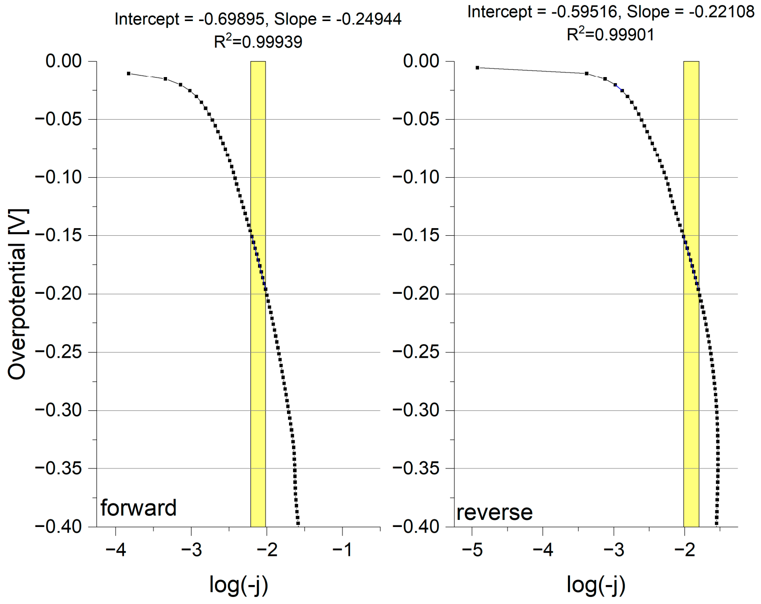

Figure S3 The linear regression applied for the determination of exchange current density $j_{0,2}$ for a base electrolyte for forward and reverse scan for overpotentials from -150 mV to -200 mV.

Furthermore, exchange current density $j_{0,2}$ that corresponds to the reduction of Cu^{2+} to Cu^{+} was obtained by fitting the linear part of the Tafel equation for cathodic processes that is valid for high negative values of overpotentials (η < 0.1 V):

$$\eta = a - b \log - j_{0,2}, \tag{3}$$

where: η – overpotential,

$$a = 2.303 \frac{RT}{\alpha_{0,2} F} \log j_{0,2} \tag{4}$$

$$b = 2.303 \frac{RT}{\alpha_{0,2} F} = 2.303 \frac{0.0257}{\alpha_{0,2}} \tag{5}$$

and $\alpha_{0,2}$ – transfer charge coefficient for Cu^{2+} reduction
Finally, after reduction and simplification equation (3) the exchange current density $j_{0,2}$ can be calculated from follow equation:

(6)

$$j_{0,2} = 10^{\frac{a}{b}} = 10^{\frac{intercept}{slope}}$$

where: a and b parameters are expressed by equations (4) and (5), while intercept and slope were calculated by linear fitting regression as it shown in Figure S9.1 For example, for base solution for forward scan (Figure S9, left side) intercept is equals 0.699 while slope 0.249, respectively. After putting these values to equation (6) the exchange current density $j_{0,2}$ is equal 15.8 mA.

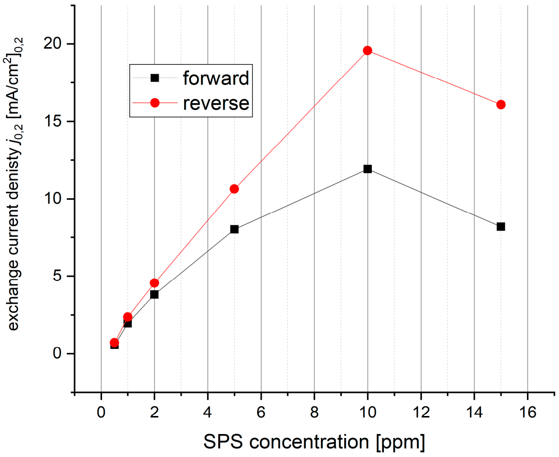

Figure S4 Distribution exchange current density $j_{0,2}$ as a function of SPS injected to the copper electroplating solution containing PEG/Cl.

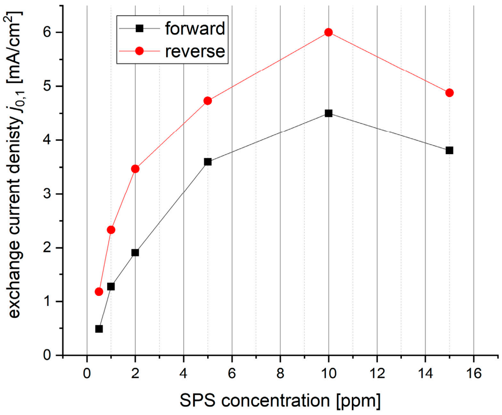

Figure S5 Distribution exchange current density $j_{0,1}$ as a function of SPS injected to the copper electroplating solution containing PEG/Cl

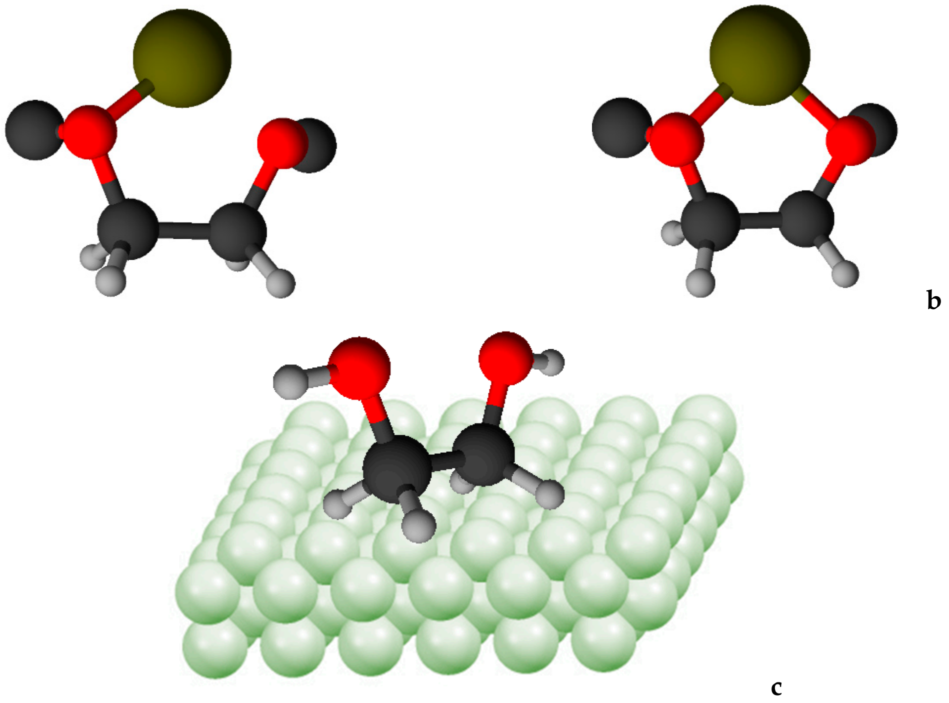

Figure S6. The proposed, simplified molecular model of $C_3H_6OCu^+$ (a) and $C_2H_4O_2Cu^+$ (b) fragments and hydrophobic interaction of PEG with chloride adlayer (c).

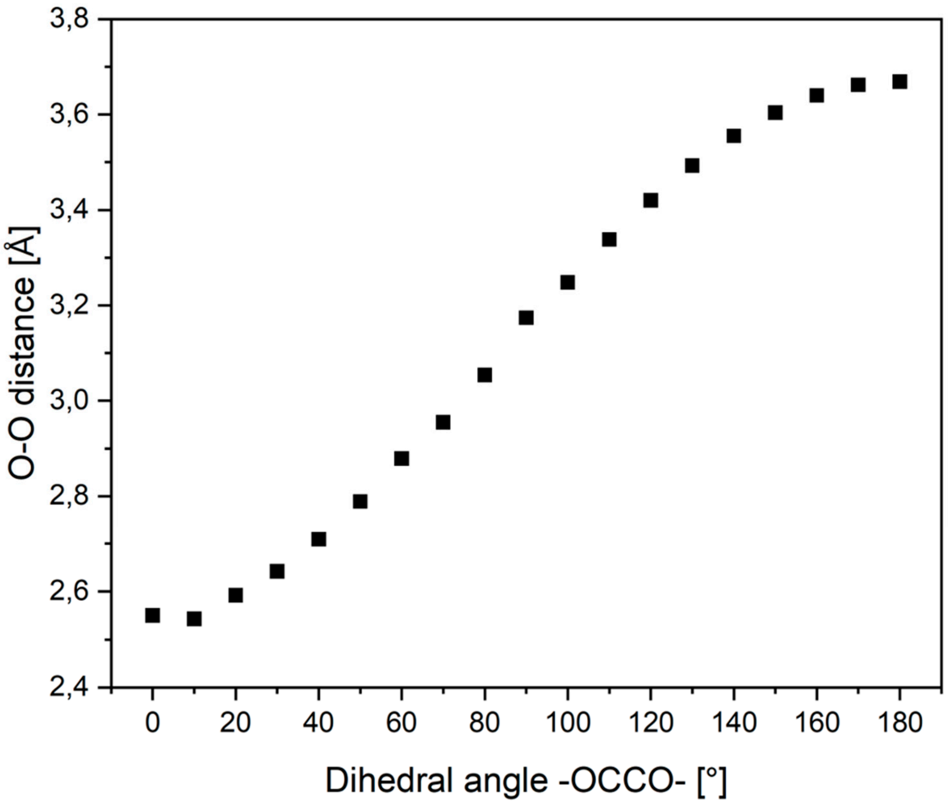

Figure S7 The O-O distance as a function of dihedral angle -OCCO- for monomer unit of PEG.

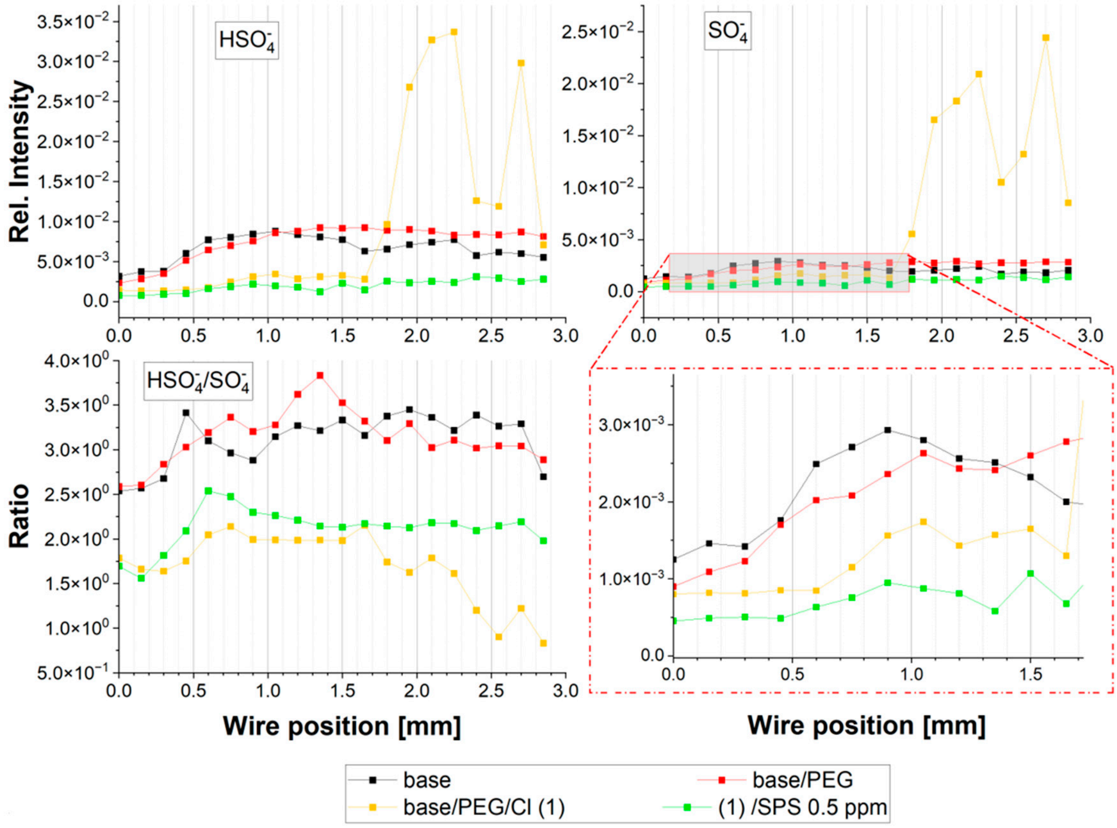

Figure S8 Distribution of intensity hydrosulfate and sulfate ions along the wire position for: base solution, base+PEG, base/PEG/Cl and base/PEG/Cl/SPS0.5 ppm

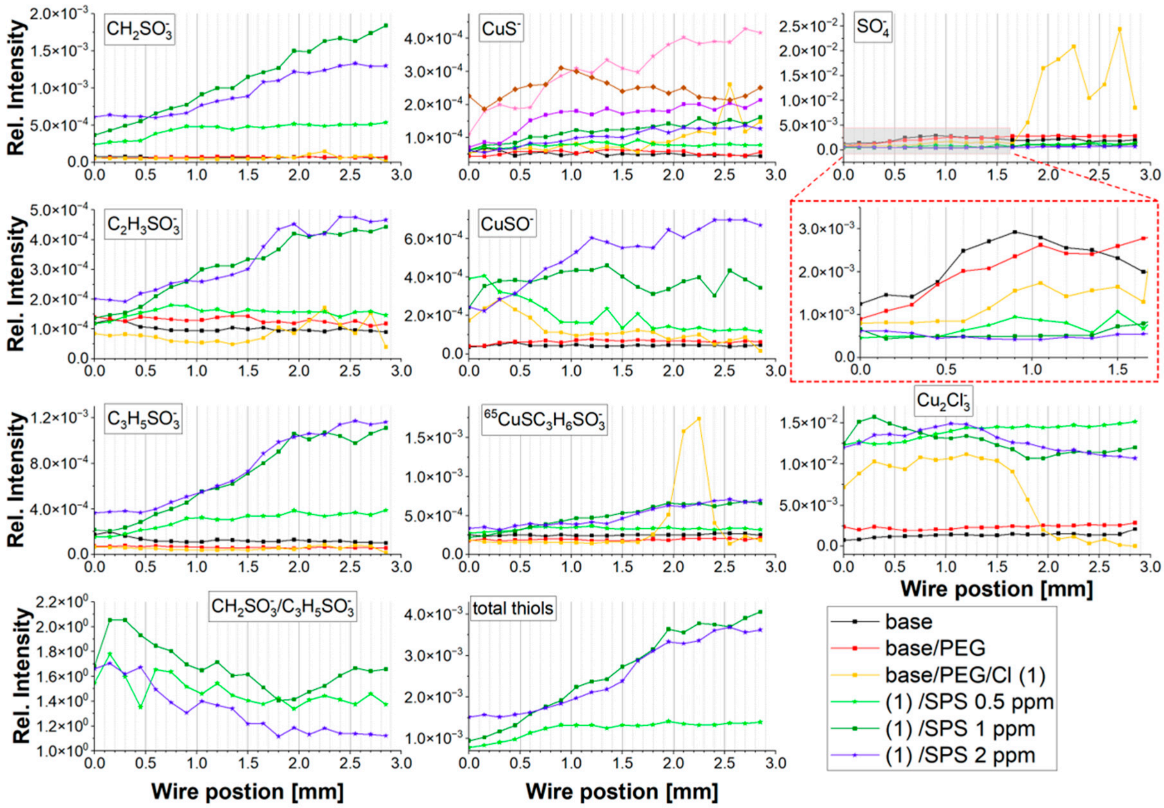

Figure S9 Intensity distribution of negative fragments identified in the TOF-SIMS mass spectra for samples containing SPS at concentration up to 2 ppm.
